# BTG2-deficient mast cells remodel the tumor and tumor-draining lymph node microenvironment leading to chemotherapy resistance in breast cancer

**DOI:** 10.3389/fimmu.2025.1562700

**Published:** 2025-04-17

**Authors:** Xiaoqian Zhang, Jiayi Wang, Ziyu Liu, Jiahuai Wen, Mengling Kang, Chen Fang, Liping Ren

**Affiliations:** ^1^ Department of Breast Oncology, The Second Affiliated Hospital of Guangzhou University of Chinese Medicine, Guangzhou, China; ^2^ Department of Breast Oncology, Breast Disease Specialist Hospital of Guangdong Provincial Hospital of Chinese Medicine, Guangzhou, Guangdong, China; ^3^ Breast Tumor Center, Sun Yat-Sen Memorial Hospital, Sun Yat-Sen University, Guangzhou, China; ^4^ Department of Rheumatology, The Sixth Affiliated Hospital, Sun Yat-sen University, Guangzhou, Guangdong, China

**Keywords:** metastatic lymph nodes, mast cells, BTG2 gene, anti-tumor immunity, αSMA^+^ fibroblasts

## Abstract

**Background:**

Breast cancer is currently the most frequently diagnosed malignancy worldwide, with chemotherapy resistance being a major contributor to breast cancer-related mortality and distant metastasis. The role of lymph nodes as the initial site of immune defense remains controversial, particularly regarding whether complete dissection or preservation is necessary during breast cancer surgery.

**Methods:**

We performed single-cell RNA sequencing (scRNA-seq) on cells derived from metastatic tumor draining lymph nodes and tumor tissue of four breast cancer patients exhibiting either sensitivity or resistance to neoadjuvant chemotherapy (NAC).

**Results:**

Mast cells with low BTG2 expression were identified in the metastatic lymph nodes and *in situ* tumor of the NAC-resistant group. Mast cells with low BTG2 expression have enhanced migratory capacity and are preferentially recruited to lymph nodes by cytokines such as CCL5, secreted by tumor cells during metastasis. Mechanistically, the mast cells with low BTG2 suppress anti-tumor immunity by inducing Treg cell production through IL-2 secretion, particularly within tumor-draining lymph nodes. Furthermore, the mast cells with low BTG2 promote NAC resistance by inducing fibroblast precursor cells to differentiate into α-SMA-positive fibroblasts via the Tryptase-PAR-2-pERK signaling pathway, leading to excessive collagen fiber production. Finally, we demonstrated that combining radiotherapy upregulating the expression of BTG2 in mast cells with chemotherapy enhances therapeutic efficacy in a murine model.

**Conclusions:**

This study highlights the immunoregulatory role of mast cells in the breast cancer tumor microenvironment and establishes a link between BTG2 expression in mast cells and neoadjuvant chemotherapy response. These findings provide a foundational basis for preserving functional lymph nodes and optimizing combined radiotherapy treatment strategies.

## 1 Introduction

Breast cancer has become the most common malignant tumor in the world, accounting for nearly 30% of female cancers (1). Although current treatments include surgery, chemotherapy, radiotherapy, targeted therapy and immunotherapy, an optimal immune response is critical to the efficacy of many traditional cancer treatments (2). Lymph nodes are the first stop of immunity, especially after they receive tumor cells, their functions become complex. On the one hand, the antigen presentation response in the lymph nodes is strengthened and more anti-tumor immunity is activated. On the other hand, the tumor itself or by inducing other cells produces an immunosuppressive effect on the lymph nodes (3). Lymph node dissection is a traditional surgical procedure, but more and more clinical follow-up data show that patients with partial lymph node preservation during surgery have a better prognosis (4). This requires a more accurate judgment of the functional type of the patient’s lymph nodes to determine the number and type of lymph nodes to retain. Therefore, combined strategies of chemotherapy, radiotherapy, immunotherapy, and biomarker-guided conventional treatments in the TME pave the way for the next generation of precision medicine (5, 6).

Mast cells (MCs) are tissue-resident immune cells that play an important role in chronic inflammation-related diseases such as cancer. Because MCs can infiltrate solid tumors and promote or restrict tumor growth, it has been proposed that MCs may be polarized into pro- or anti-tumor phenotypes and remains a challenging area of research (7). The ability to invade solid tumors is considered an important marker associated with better or worse prognosis, depending on the type and tumor stage (8). In some solid tumors, MCs are detected in the intratumoral region, whereas in other solid tumors they are preferentially located in the peritumoral region. Interestingly, the presence of peritumoral MCs appears to indicate a poor prognosis, whereas their intratumoral location is associated with both favorable and unfavorable prognosis. Mast cells constitute the major population of immune cells and promote tumor progression through immune complexes, cytokines, and immune checkpoints (9). However, a growing body of research shows that the density of mast cells and Th1 cells is closely associated with improved patient survival (10). More importantly, clinical trials suggest that depletion of total mast cells may adversely affect antitumor immunity (11). Taken together, these conflicting observations strongly suggest that mast cells in tumors consist of distinct subpopulations with opposing functions (12). However, a detailed understanding of the cellular diversity of mast cells in tumors is limited, and the underlying mechanistic switches of their pro- or anti-tumor phenotypes remain largely unknown.

Here, we provide comprehensive single-cell RNA profiling of paired neoadjuvant chemotherapy-resistant and sensitive mast cells in tumor-infiltrating and metastatic lymph nodes in clinical samples. We identify a distinct mast cell subset emerging after chemotherapy and reveal how this subset affects anti-tumor immunity and contributes to chemotherapy resistance.

## 2 Materials and methods

### 2.1 Human specimens

This study included four patients who underwent surgery at Department of Breast Oncology, the Guangdong Provincial Hospital of Chinese Medicine. In total, eight fresh surgical specimens (four primary tumors and four paired metastatic lymph nodes) were sequenced and incorporated in further analyses. The metastasis of lymph nodes was confirmed by pathologists through both cytological detections during the surgery and the paraffin section after surgery. All procedures involving human specimens were approved by the Institutional Review Board of Guangdong Provincial Hospital of Chinese Medicine, and written informed consent was obtained from all patients.

### 2.2 Cell lines and cell culture

The murine breast cancer cell line E0771, HFL-1 cell lines were purchased from ATCC. The mouse mast cell line P815 was purchased from Cell Bank/Stem Cell Bank, Chinese Academy of Sciences (13). E0771 breast cancer cells were cultured in Roswell Park Memorial Institute (RPMI) 1640 medium supplemented with 10% fetal bovine serum (FBS, Gibco) and 1% pen-strep antibiotic (Thermo). HEK293T cells were cultured in Dulbecco’s Modified Eagle Medium (DMEM) (Thermo Fisher) supplemented with 10% FBS and 1% penicillin-streptomycin. All cell lines were tested and authenticated. All cells were cultured at 37°C in a humidified atmosphere containing 5% CO₂. The cell lines were Mycoplasmafree and authenticated by PCR analysis monthly. In some experiments, HFL-1 cells were pre-treated with PAR2 inhibitors: GB83 (Cat# 1252806-86-2, Sigma) or FSLLRY-NH₂ (Cat# 245329-02-6, Sigma) (14). Mast cells were treated with 5 mM N-Acetyl-L-cysteine (Cat# A7250, Sigma) (15).

### 2.3 Tumor sample handling and dissociation to a single-cell suspension for scRNA-seq

Tumor and lymph node specimens were surgically excised by a skilled physician to ensure that the tumor samples contained a mix of neoplastic, stromal, and adjacent non-cancerous tissues, while the lymph node samples included both neoplastic and lymphoid tissues with a complete cross-section. Fresh tissue samples were washed twice with chilled RPMI 1640 medium containing 0.04% fetal bovine serum (BSA, Gibco). For scRNA-seq, the tissue was cut into approximately 1 mm³ pieces and subjected to enzymatic digestion using 10 mL of digestion medium containing 0.1% collagenase type I and collagenase type III (Worthington Biochemical), 0.05% hyaluronidase type I-S (Sigma-Aldrich), and 0.002% DNase I (Sigma). The digestion was carried out for 45 minutes at 37°C in a shaking water bath. After digestion, the tissue mixture was filtered through a 100-µm nylon mesh and centrifuged at 300×g for 10 minutes to collect a single-cell suspension. To remove red blood cells, the suspension was treated with a red blood cell lysis solution (Cat# 555899, BD) for 2 minutes, followed by centrifugation at 300×g for 10 minutes. The supernatant was discarded, and the resulting cell pellet was washed twice with RPMI 1640 medium containing 0.04% BSA. Finally, the cells were resuspended in sorting buffer (PBS supplemented with 0.04% FBS). Cell concentration and viability were assessed by Luna cell counter.

### 2.4 scRNA-seq and reads processing

The concentration of each fresh cell suspension was adjusted to 700-1200 cells/µL. Singleron platform process raw reads to generate gene expression matrices with the CeleScope pipeline.

### 2.5 Quality control and batch effect correction of scRNA-seq data

The UMI count matrix was processed using the Seurat R package (version 4.3.0). To eliminate low-quality cells and potential multiplet captures, a key concern in microdroplet-based experiments, cells were filtered based on the number of UMIs and genes. Specifically, cells with UMI/gene counts deviating more than three standard deviations from the mean (assuming a Gaussian distribution) were excluded. Additionally, cells with more than 5% of their total gene counts derived from mitochondrial genes were considered of low quality and discarded after visual inspection of the distribution. To further ensure cellular integrity, we retained cells with ≥1,000 unique molecular identifiers (UMIs), ≥200 detected genes, and <5% mitochondrial reads. Doublets were removed using scDblFinder, and cell cycle stages were assigned to assess technical artifacts. After applying these quality control filters, a total of 26,855 single cells remained for further analysis. Library size normalization was performed using the NormalizeData function in Seurat, which applied the “LogNormalize” method. This method scaled the gene expression by the total expression per cell, multiplied by a default scaling factor of 10,000, followed by log-transformation. The normalized expression profiles of all samples were then combined using the merge function in R (v4.1.1). To correct for batch effects in the single-cell RNA sequencing data, mutual nearest neighbors were calculated using the batchelor R package. Additionally, batch effects were mitigated using Harmony integration with PCA (first 30 PCs) and batch_id as the grouping variable, validated by UMAP visualization and cluster homogeneity metrics. Our approach prioritizes biological fidelity while removing technical noise, aligning with best practices for single-cell analysis (16, 17). These revisions ensure transparency and robustness in our workflow (18).

### 2.6 Transwell assays

The transwell migration assays were conducted with Corning Transwell Inserts (8.0 µm). For the transwell migration assay, 1.5×10^4^ transfected mast cells suspended in 50 µL serum-free medium were placed in the upper chamber and 600 µL medium (10% FBS) containing 50 ng/ml CCL5 was filled in the lower compartment. The cells were incubated at 37°C for 12 h. The successfully translocated cells were fixed with 4% paraformaldehyde and stained with 0.1% crystal violet, and counted in four randomly chosen fields (200×) under a microscope.

### 2.7 Animal experiments

We injected 5×10^5^ E0771 cells into the fat pads of six-week-old female C57/BL6 mice. In certain experiments, mast cells (transduced with or without lentiviral vectors containing BTG2 shRNA or BTG2 expression) were injected into mouse tail vein. Once the syngeneic grafts became palpable, 6-8 mice per group were randomly assigned to different treatment regimens. PBS, Adriamycin (5 mg/kg) were administered intraperitoneally on a weekly basis. Tumor volumes were measured every 3 days using the formula: volume=(width^2^×length)/2. Mice were euthanized when tumors reached an approximate size of 1,200 mm^3^ in volume. Tumor, lymph node and Mouse Bone Marrow Cells suspensions were prepared following previously described protocols (19). Tissue was washed with PBS, minced into 1-2 mm^3^ pieces, and incubated in DMEM with 5% FBS, 0.1% collagenase I (Worthington Biochemical), 0.1% collagenase IV (Worthington Biochemical), and 0.002% DNase I (Sigma) for 30 minutes at 37°C with continuous shaking. During digestion, tissue and pieces were vortexed every 15 minutes. The cell suspensions were filtered through a 100 µm strainer, washed twice with PBS, and used for further experiments. All animal procedures were approved by the Institutional Review Boards and Animal Care and Use Committees of Sun Yat-Sen University.

In some experiments, mice were treated with 200 ug twice weekly anti-IL-2 (Cat# BE0042, BioXCell). Control mice received equal amounts of isotope control antibodies (Cat# BE0091, BioXcell) (20, 21).

### 2.8 qRT-PCR

Total RNA was obtained from cells using Trizol (Takara) following the manufacturer’s protocol. cDNA was synthesized using a reverse transcription kit (Thermo). The qRT-PCR was performed with cDNA as template using the 2X SYBR Green Master Mix (Thermo).

The forward primer of GAPDH was 5’-ATCACCATCTTCCAGGAGCGA-3′ and reverse was 5’-CCTTCTCCATGGTGGTGAAGAC-3′.

The forward primer of BTG2 was 5′- GCACTCACAGAGCACTACAA-3′ and reverse was 5′-TGCGGTAGGACACCTCATA-3′.

The forward primer of COL1A1 was 5′-GATTCCCTGGACCTAAAGGTGC-3′ and reverse was 5′- AGCCTCTCCATCTTTGCCAGCA-3′.

The forward primer of COL1A2 was 5′- CCTGGTGCTAAAGGAGAAAGAGG-3′ and reverse was 5′- ATCACCACGACTTCCAGCAGGA-3′.

The forward primer of COL3A1 was 5′- TGGTCTGCAAGGAATGCCTGGA-3′ and reverse was 5′- TCTTTCCCTGGGACACCATCAG-3′.

### 2.9 Flow cytometry

Suspended cells were stained for Live/Dead Fixable Viability Dye: FVD-eFluor780,(Cat# 65-0865-18, eBioscience) in PBS for 15 min at RT to distinguished live and dead cells. To block non-specific binding, mouse and human cells were incubated with FcR blocking reagent for mice (Cat# 130-092-575, Miltenyi Biotec) and humans (Cat# 130-059-901, Miltenyi Biotec) respectively. For intracellular cytokine staining, cells were re-stimulated with E0771 cells line in the presence of a protein transport inhibitor cocktail containing Brefeldin A (Cat# 420601, Biolegend). The monoclonal antibodies are as follows: anti-human CD45 (Cat# 304006, Biolegend), anti-mouse CD117 (Cat# 135123, Biolegend), anti-human CD117 (Cat# 375211, Biolegend), anti-mouse CD45 (Cat# 147717, Biolegend), anti-mouse CD8a (Cat# 100729, Biolegend), anti-mouse CD4 (Cat# 130308, Biolegend), anti-mouse Perforin (Cat# 154303, Biolegend), anti-mouse IFN-γ (Cat# 505807, Biolegend), anti-mouse FOXP3 (Cat# 25-5773-82, Thermo), anti-human FOXP3 (Cat# 25-4776-42, Thermo), anti-human CD4 (Cat# 344604, Biolegend). Intracellular cytokine staining was performed using an Intracellular Fixation and Permeabilization kit (Cat# 88-8824, eBioscience) according to the manufacturer’s instructions. For some experiments, Propidium Iodide (Cat#00-6990-50, eBioscience) was used to detect the death of tumor cells. The samples were analyzed by the Beckman CytoFLEX Flow cytometer. Data were analyzed by FlowJo software.

### 2.10 Knockdown of BTG2 in mast cells

To knock down BTG2, shRNA targeting human BTG2 (5′‐CACTCACAGAGCACTACAAAC‐3′) or mouse BTG2 (5′‐GGACGCACTGACCGATCATTA‐3′) was constructed by Gene Pharma (Shanghai, China). The indicated shRNA was cloned into lentivirus vector pLKO.5 puro (Addgene) and confirmed via sequencing analysis. Lentivirus was produced by transient co‐transfection of HEK293T cells with a lentivirus vector, packaging plasmid, psPAX2, and pMD2.G using PEI MAX (Polysciences) (22). Viral supernatant was collected at 48 and 72 h after transfection and was concentrated by ultracentrifugation (23). BTG2 siRNA (Cat#, sc-44818, Santa Cruz Biotechnology) were utilized for knockdowns, as described previously.

### 2.11 Preparation of human blood-derived mast cells

Peripheral blood mononuclear cells (PBMC) were isolated by density gradient centrifugation using Ficoll-Paque PLUS (Cytiva) according to the manufacturer’s protocol. The isolated PBMCs were washed three times with phosphate-buffered saline (PBS) and resuspended in RPMI-1640 medium supplemented with 10% fetal bovine serum (FBS), 1% penicillin-streptomycin, and 2 mM L-glutamine. PBMCs were cultured at a density of 1×10^6^ cells/mL in complete RPMI-1640 medium supplemented with 100 ng/mL recombinant human stem cell factor (SCF), 30 ng/mL interleukin-3 (IL-3) and 100 ng/mL interleukin-6 (IL-6) (PeproTech, USA). The medium was replaced every 3-4 days, and fresh cytokines were added during each medium change. Cells were maintained at 37°C in a humidified atmosphere containing 5% CO_2_. The culture process was continued for an additional 4-6 weeks, with periodic medium changes and cytokine replenishment.

### 2.12 Human mast cell-T cell co-culture

Human naive CD4 T cells were isolated from PBMC, which were donated from healthy volunteer donors as aforementioned. naive CD4 T cells were obtained through MicroBeads (Stemcell; 19555). These naive CD4 T cells (2-3×10^6^ cells) were co-cultured with and without peripheral blood-derived cultured mast cells (5×10^5^ cells) in the presence and absence of 100 ng/ml rhIL-33 for 4 days. Foxp3 expression in CD4^+^CD25^+^ T cells was analyzed by FlowJo software.

### 2.13 Human mast cell–fibroblast cell co-culture

HFL-1 cells are cultured in F-12K medium supplemented with 10% fetal bovine serum (FBS) and 1% penicillin-streptomycin under a humidified atmosphere at 37°C with 5% CO_2_. For co-culture experiments, mast cells and HFL-1 cells labeled with Cell Trace Deep red were seeded in the same culture system, with a density of 1×10^6^ mast cells per well and 2×10^5^ fibroblasts per well. Co-cultures were maintained for up to 24 hours. The fibroblast activation was evaluated by α-smooth muscle actin (α-SMA) expression and measuring collagen production.

### 2.14 Preparation of bone marrow-derived cultured mast cells (BMCMCs)

BMCMCs were obtained by culturing mouse femoral BM cells in DMEM medium (Sigma), supplemented with 10% fetal bovine serum (Gibco), 1% Penicillin/Streptomycin (Gibco), 100 ng/ml recombinant mouse stem cell factor (SCF) and 30 ng/ml mouse IL-3 (both from Peprotech) for 4 to 6 weeks.

### 2.15 Patient derived xenograft (PDX) experiments

Humanized mice were generated from NSG mice. Briefly, 3-4 week female NSG mice were subjected to 2 cGy total body irradiation 12 hr before tail vein injection with 2×10^5^ CD34+ cells isolated from PBMCs by direct CD34 Cell Isolation Kit (Cat# 130-046-702, Miltenyi Biotec). To establish patient-derived xenografts, primary tumor specimens were collected from breast cancer patients who underwent tumor resection at Department of Breast Oncology, the Guangdong Provincial Hospital of Chinese Medicine between 2021 and 2023. Eight-week-old NOD-SCID mice under pathogen-free conditions were used for patient-derived xenograft transplantation. Briefly, a small incision was made on the abdomen of Humanized mice to reveal the mammary gland and primary breast tumor samples were minced into 1 mm^3^ sized fragments and injected directly into the fourth pair of mammary fat pads (24). The primary breast cancer samples were successfully engrafted into NSG mice to generate PDX models, with tumor growth ≥50 mm³ confirmed within 12 weeks and validated by histopathological analysis of human pan-cytokeratin staining.

### 2.16 Reagents and antibodies

Protein was extracted from the cells with RIPA (Millipore) or IP buffer (Thermo Fisher Scientific) containing proteinase inhibitor cocktail (Cat# 78446, Thermo Fisher Scientific). To evaluate p-FAK in mast cells, RIPA (Millipore) containing Halt™ Protease and Phosphatase Inhibitor Single-Use Cocktail (Cat# 78442, Thermo Fisher Scientific) were used to lysis cells. Total protein was quantified using a Pierce BCA Protein Assay Kit (Cat# 23225, Thermo Fisher Scientific). Equal amounts of lysates were fractionated by 10% SDS-PAGE and electrotransferred to PVDF membranes (Cat#03010040001, Roche). After blocking with TBS/0.05% Tween-20/5% BSA, the membranes were probed with first antibodies listed as below: rabbit anti-human p-FAK (Cat# 3283, Cell Signaling Technology; 1:1000), rabbit anti-human FAK (Cat# 3285, Cell Signaling Technology; 1:1000), rabbit anti-human p-ERK1/2 (Cat# ab201015, abcam; 1:1000), rabbit anti-human ERK1/2 (Cat# ab184699, abcam; 1:1000), rabbit anti-human Src (Cat# 2108S, Cell Signaling Technology; 1:1000), GAPDH (Cat# HRP-60004, Proteintech; 1:10,000), rabbit anti-human COL1 (Cat #72026, Cell Signaling Technology; 1:1000), rabbit anti-human COL3 (Cat #98908, Cell Signaling Technology; 1:1000), rabbit anti-human αSMA (Cat #19245, Cell Signaling Technology; 1:1000).

### 2.17 Statistical analysis

Every experiment was repeated at least three times. All data was analyzed using Graphpad prism 10.0 and presented as mean ± standard deviation (SD) or as indicated. The data was analyzed using two-tailed Student’s t-test for average differences. *P* values ≤ 0.05 was considered to be statistically significant.

## 3 Results

### 3.1 Breast cancer patients with chemoresistance exhibit a significantly higher density of mast cells in both primary tumor sites and draining lymph nodes

**Figure 1:**
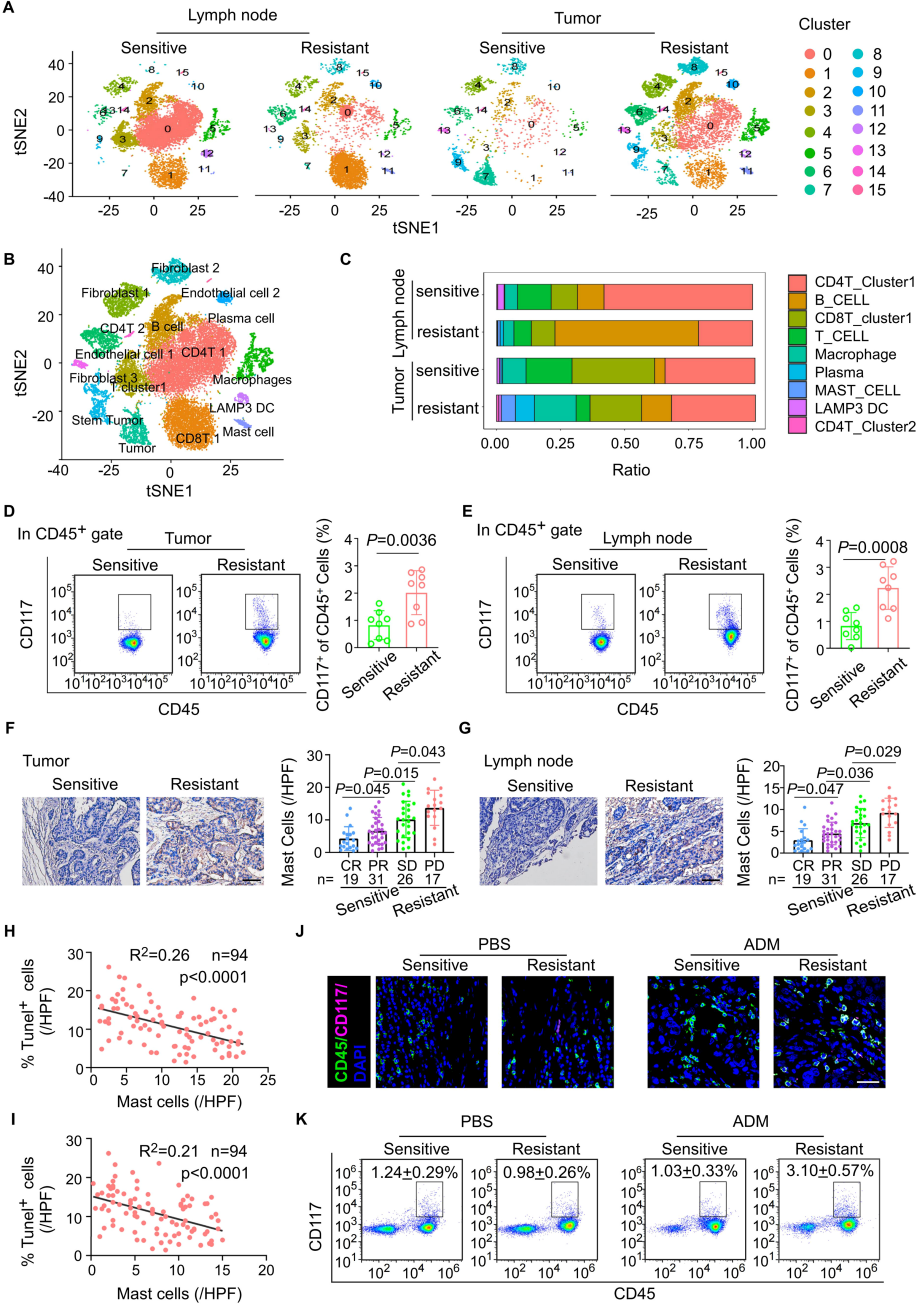
Breast cancer patients with chemoresistance exhibit a significantly higher density of mast cells in both primary tumor sites and draining lymph nodes. **(A)** t-SNE plots of cells from tumor and paired lymph node tissue of four breast cancer patients, showing 16 clusters in each plot. Each cluster is shown in a different color. **(B)** t-SNE plot of cells profiled in the present study colored by major cell types. **(C)** Based on single-cell sequencing results, the proportions of major immune cell types within the CD45^+^ subpopulation are displayed across different sample groups. **(D)** Flow cytometric analysis of the mast cells from breast cancer tissues after NAC. Representative plots (left) and quantitation (right) are shown. (n = 8). **(E)** Flow cytometric analysis of the mast cells from tumor draining lymph nodes (TLNs) of patients with breast cancer after NAC. Representative plots (left) and quantitation (right) are shown. (n = 8). **(F)** The mast cells in breast cancer tissues with different therapeutic responses after NAC. pCR, pathological complete response, n = 19; PR, partial response, n = 31; SD, stable disease, n = 26; PD, progressive disease, n = 17. **(G)** The mast cells in draining lymph node with different therapeutic responses after NAC. pCR, pathological complete response, n = 19; PR, partial response, n = 31; SD, stable disease, n = 26; PD, progressive disease, n = 17. **(H)** The correlation between the abundance of mast cells in tumor and the infiltration of Tunel^+^ tumor cells in BC tissues post-neoadjuvant chemotherapy (n = 94). Pearson’s correlation coefficient (R^2^) and p values were determined by two-tailed Pearson correlation test. **(I)** The correlation between the abundance of mast cells in lymph node and the infiltration of Tunel^+^ tumor cells in BC tissues post-neoadjuvant chemotherapy (n = 94). Pearson’s correlation coefficient (R^2^) and p values were determined by two-tailed Pearson correlation test. **(J, K)** Tumor tissues from breast cancer patients, including those who are chemotherapy-sensitive and chemotherapy-resistant, are implanted into immunodeficient NSG (NOD-SCID gamma) mice. **(J)** Immunofluorescent quantitation of CD117 and CD45 co-expressing cells (mast cells) in tumor from PBS- or ADM-treated sensitive or resistant PDX syngrafts. **(K)** Flow cytometric analysis of CD117 and CD45 co-expressing cells (mast cells) in TLN from PBS- or ADM-treated sensitive or resistant PDX syngrafts.

To investigate the differences in cellular composition and characteristics between neoadjuvant chemotherapy-resistant and chemotherapy-sensitive breast cancer patients, we performed single-cell RNA sequencing (scRNA-seq) on paired tumor and metastasis-positive draining lymph node samples from two patients in each group (**Supplementary Figures S1A, B**). The scRNA-seq analysis revealed a significantly higher abundance of mast cells in both the tumors and draining lymph nodes of the neoadjuvant chemotherapy-resistant group compared to the neoadjuvant chemotherapy-sensitive group (**Figures 1A–C**). To validate this observation, we further assessed the percentage of CD117^+^ CD45^+^ cells in paired tumor tissues and paired TLN tissues isolated form eight patients with resistant or sensitive to NAC. Intriguingly, we found that mast cells, CD117^+^ CD45^+^ cells, were enriched in the samples with resistant to neoadjuvant chemotherapy, both tumor tissues and TLN tissues (**Figures 1D, E**). Consistently, IHC staining of the both tumor tissues and TLN tissues obtained following neoadjuvant chemotherapy demonstrated abundant mast cells in in the resistant to neoadjuvant chemotherapy group (**Figures 1F, G**). Moreover, in another cohort of 94 breast cancer patients undergone NAC, we demonstrated that the density of mast cells in both the tumor stroma and TLN tissue correlated with reduced number of Tunel^+^ tumor cells, which was associated with chemotherapeutic responsiveness (**Figures 1H, I**).

To further explore the relationship between mast cells and the efficacy of neoadjuvant chemotherapy, we utilized a patient-derived xenograft (PDX) mouse model. Tumor tissues from breast cancer patients with varying responses to neoadjuvant chemotherapy were implanted into humanized NSG mice, which were subsequently treated with doxorubicin (ADM) every three days to evaluate chemotherapy response (**Supplementary Figure S1C**). Consistent with clinical observations, PDX mice derived from chemotherapy-resistant tumors exhibited poorer responses to chemotherapy than those derived from chemotherapy-sensitive tumors, (**Supplementary Figures S1D-F**). Notably, the number of mast cells in both primary tumor sites and draining lymph nodes was significantly elevated in chemotherapy-resistant PDX mice compared to the sensitive group (**Figures 1J, K**). In summary, these findings establish a significant association between mast cell abundance and NAC resistance in breast cancer, providing insight into potential mechanisms underlying treatment failure.

### 3.2 The mast cells are characterized by low expression of BTG2, and exogenous infusion of BTG2sh mast cells will reduce the efficacy of chemotherapy

**Figure 2:**
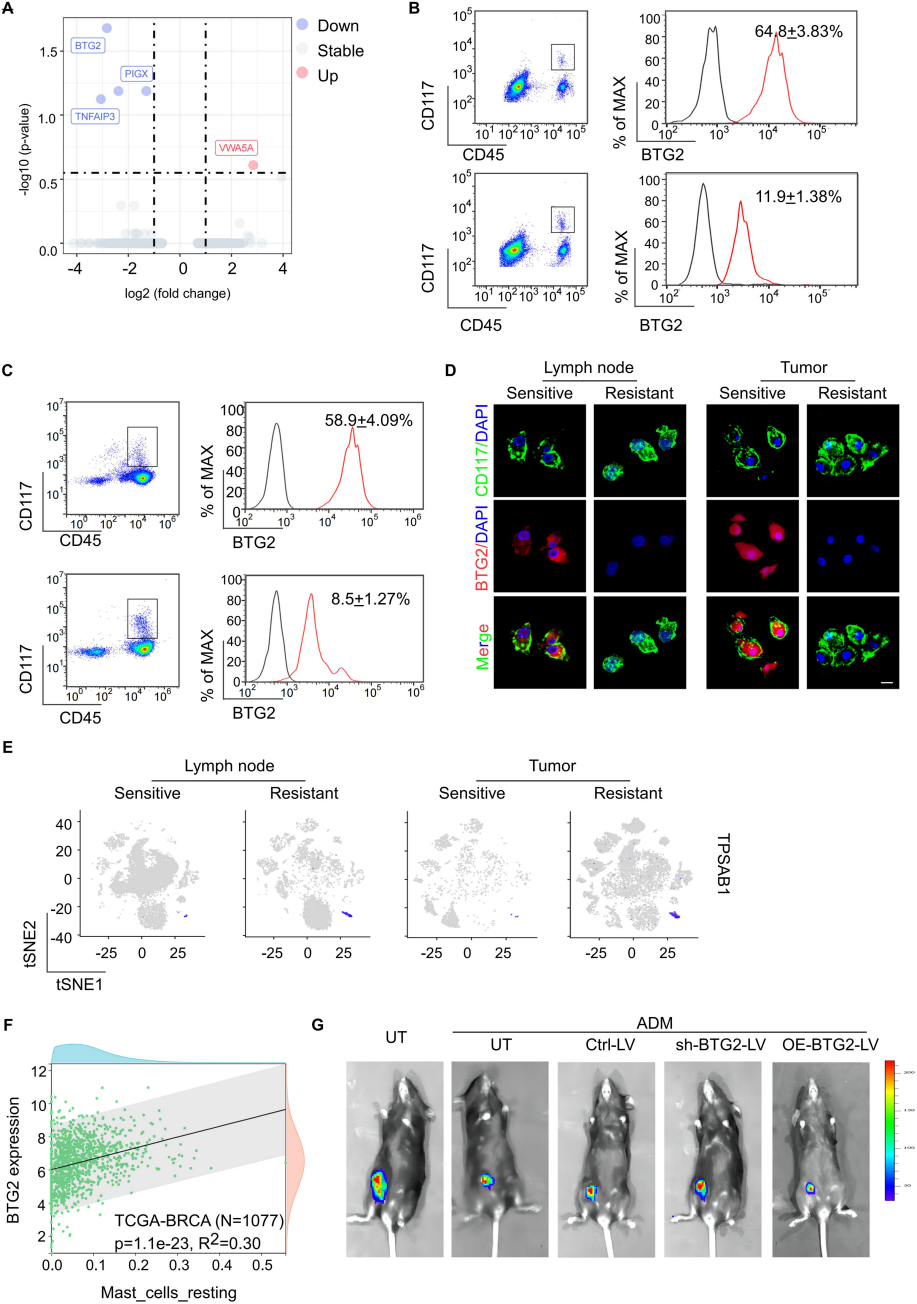
The mast cells are characterized by low expression of BTG2, and exogenous infusion of BTG2sh mast cells will reduce the efficacy of chemotherapy. **(A)** Differential gene expression analysis of mast cell between sensitive or resistant to NAC group. The volcano plot displays log2 fold change (x-axis) versus -log10 *p*-value (y-axis) for all analyzed genes. Genes with a |log2 fold change| > 1 and a p-value < 0.05 are highlighted in red (upregulated) and blue (downregulated). The horizontal dashed line represents the significance threshold (*p* < 0.05), while the vertical dashed lines indicate the fold change thresholds. **(B)** Flow cytometric analysis of the expression of BTG2 in mast cells isolated from tumor tissue of post-NAC BC patients between sensitive or resistant group (mean ± s.d., n = 5). **(C)** Flow cytometric analysis of the expression of BTG2 in mast cells isolated from TDLNs of post-NAC BC patients between sensitive or resistant group (mean ± s.d., n = 5). **(D)** Representative immunofluorescence co-staining for BTG2 and CD117 in tumor-infiltrating mast cells(right) and TLN-infiltrating mast cells (left) isolated from fresh tissues of human breast cancer sensitive or resistant to NAC. **(E)** Gene overlays of TPSAB1 marker on t-SNE of single cells from tumor tissue and paired TDLNs tissue. **(F)** The scatter plot displayed the correlation between the infiltration level of resting mast cells and BTG2 gene expression is shown. Data sourced from the TCGA breast cancer database (N=1077). **(G)** Representative bioluminescent images showing the tumor growth in C57BL/6 mice inoculated with luciferase-E0771 tumor cells were immunized with PBS or ADM only or along with mast cells that were untreated (UT), transfected with ctrl-vector (Ctrl), BTG2 shRNA (shBTG2) or BTG2-expressing lentivirus (OE-BTG2).

To further investigate the molecular characteristics and the role of enlarged mast cells which are particularly prevalent in the tumor and TLN tissues of NAC-resistant patients, we identified the low expression of BTG2 as the predominant differential molecular feature in these cells (**Figure 2A**). BTG2 gene encodes a protein belonging to the BTG family, which is integral to key biological processes, including cell proliferation, differentiation, and apoptosis (25). BTG2 plays essential roles in cell cycle regulation, transcription, and DNA repair and is widely expressed across various cell types. Notably, high BTG2 expression has been associated with maintaining the quiescent state of T cells, B cells, and other immune cells (26). Using flow cytometry and immunofluorescence staining, we confirmed significantly lower BTG2 expression in both tumor tissues and TLN tissues in the NAC-resistant group (**Figures 2B–D**). The bulk RNA-seq data from the TCGA breast cancer dataset. exhibited a significant correlation between low BTG2 expression and poor prognosis to chemotherapy in a larger patient cohort by kmplot web analysis (**Supplementary Figures S2A**) (27). Consistent with previous studies, our single-cell RNA sequencing data also revealed that high BTG2 expression correlates with mast cell the quiescence status (**Figure 2E**; **Supplementary Figure S2B**). This observation was further supported by transcriptomic analysis of 1,077 breast cancer patients from The Cancer Genome Atlas (TCGA) and immune cell infiltration analysis using Timer2.0 (**Figure 2F**).

To delineate the role of BTG2 expression loss specifically in mast cells in the context of chemotherapy resistance, we modulated BTG2 expression *in vitro*. Mast cells with BTG2 knockdown or overexpression were injected into E0771 tumor-bearing mice undergoing doxorubicin (ADM) treatment (**Supplementary Figures S2C, D**). Remarkably, the infusion of BTG2-deficient mast cells reduced chemotherapy efficacy and increased lymph node metastasis in the breast cancer mouse model, compared to untreated mast cells. Conversely, infusion of mast cells overexpressing BTG2 improved chemotherapy efficacy and reduced lymph node metastasis (**Supplementary Figures S2E-G**; **Figure 2G**). These findings highlight that mast cells characterized by low BTG2 expression contribute to chemotherapy resistance, and exogenous infusion of BTG2-deficient mast cells exacerbates this resistance.

### 3.3 Mast cells with low BTG2 expression have stronger migration ability

**Figure 3:**
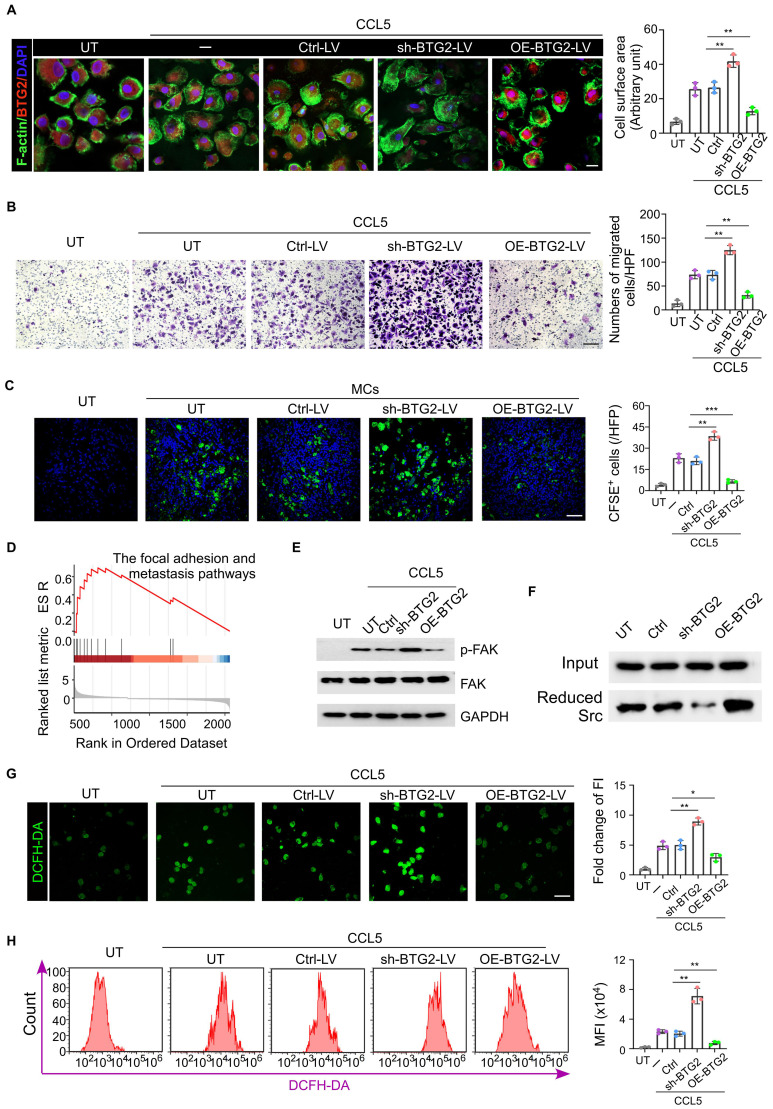
Mast cells with low BTG2 expression have stronger migration ability. **(A)** Mast cells transduced with untreat (UT) ctrl-vector (Ctrl-LV), BTG2 shRNA (shBTG2-LV) or BTG2-expressing lentivirus (OE-BTG2-LV) were treated with or without 50 ng ml^-1^ CCL5. The representative images of filopodium-like protrusions of the cells (stained with phalloidin (F-actin, green)) are shown on the left, and the quantification is shown on the right. Scale bars, 20 μm. n = 3 biologically independent experiments. Data are mean ± s.d. **(B)** Representative images of transwell assays (left) and numbers of migration cells (right) in UT or Ctrl-LV, shBTG2-LV, OE-BTG2-LV with CCL5 groups. **(C)** Representative fluorescence images for CFSE-label cells in tumor tissues of E0771 tumor-bearing mice in the presence or absence of tail vein injection of 1×10^4^ CESE-label mast cells transfected without (UT) or with ctrl, sh-BTG2 or OE-BTG2 plasmid. **(D)** GSEA enrichment analysis display genes in the focal adhesion and metastasis pathways showed significant enrichment in BTG2 low versus BTG2 high in mast cells. **(E)** Western blotting for total FAK, phosphorylated FAK in the cell extraction (CE) of mast cells transfected without (UT) or UT or Ctrl-LV, shBTG2-LV, OE-BTG2-LV with CCL5 groups. **(F)** Western blot of Reduced Src co-IP with BIAM labeling. **(G)** Representative fluorescence images for intracellular ROS stained by DCFH-DA, and the quantity of production of relative fluorescence units. **(H)** Representative flow cytometry images showed the intracellular ROS levels and the quantity of production of intracellular ROS was measured using DCFH-DA by flowcytometry among the various groups. **p < 0.01, ***p < 0.001 by Student's t-test.

There is substantial evidence that BTG2, a well-characterized tumor suppressor gene, plays a critical role in suppressing the migratory capacity of tumor cells by modulating key molecular pathways that govern cell movement and invasion (28, 29). This has led to the hypothesis that BTG2 might exert a similar effect in cells, which are involved in enrichment in tumor tissue and draining lymph nodes. Firstly through migration assays and immunofluorescence staining experiments, we observed that mast cells in the BTG2 knockdown group demonstrated significantly stronger migratory capabilities compared to the untreated group, suggesting a direct involvement of BTG2 in regulating cell migration. In contrast, mast cells that overexpressed BTG2 displayed a marked reduction in migration ability, further supporting the notion that BTG2 negatively regulates cell movement (**Figures 3A, B**). Building on these observations, we performed *in vivo* experiments involving tail vein injections of exogenous CFSE-labeled mast cells to verify with low BTG2 expression exhibited stronger migratory ability toward tumor sites and their draining lymph nodes (**Figure 3C**; **Supplementary Figure S3A**). To further investigate the molecular mechanisms underlying BTG2’s regulation of mast cell migration, we conducted Gene Set Enrichment Analysis (GSEA) on the signaling pathways of mast cells isolated from a drug-resistant cancer model using previously generated scRNA sequencing data. The GSEA results revealed that the focal adhesion pathway associated with cell migration, were significantly upregulated in mast cells from the drug-resistant group (**Figure 3D**).

Previous studies have shown that BTG2 inhibits tumor migration ability by regulating the production of mitochondrial reactive oxygen species (ROS) (29). BTG2 reduces ROS levels, thereby reducing the oxidative state of Src, a key kinase involved in phosphorylating focal adhesion kinase (FAK). Western blot (WB) analysis showed FAK phosphorylation in mast cells with BTG2 knockdown group (**Figure 3E**; **Supplementary Figure S3B**). Previous studies have shown that its specific mechanism is that BTG2 reduces the production of mitochondrial ROS and reduces Src, leading to the phosphorylation of FAK. Western blot experiment observed more phosphorylation of FAK in mast cells in the BTG2 knockdown group, and BIMA labeling experiments detected more reduced Src in the BTG2 over-expression group (**Figure 3F**). DCFH-DA, as a marker for ROS detection, was more stained in mast cells isolated *in situ* from chemotherapy-resistant breast cancer patients, and showed a higher degree of staining in mast cells of the BTG2 low expression group in *in vitro* cell experiments (**Supplementary Figures S3C**; **Figures 3G, H**). The ROS scavenger N-acetylcysteine (N-ac) could abolish the increase of mast cell migration and FAK phosphorylation due to the knock-down of BTG2 (**Supplementary Figures S3D, E**). The above data indicate that BTG2 indirectly reduces the oxidative activation of Src by reducing ROS levels, thereby inhibiting Src-mediated FAK phosphorylation and further inhibiting the migration ability of mast cells.

### 3.4 Mast cells with low BTG2 expression induce the differentiation of naive CD4 T cells into Treg cells through IL-2, suppressing tumor immunity

**Figure 4:**
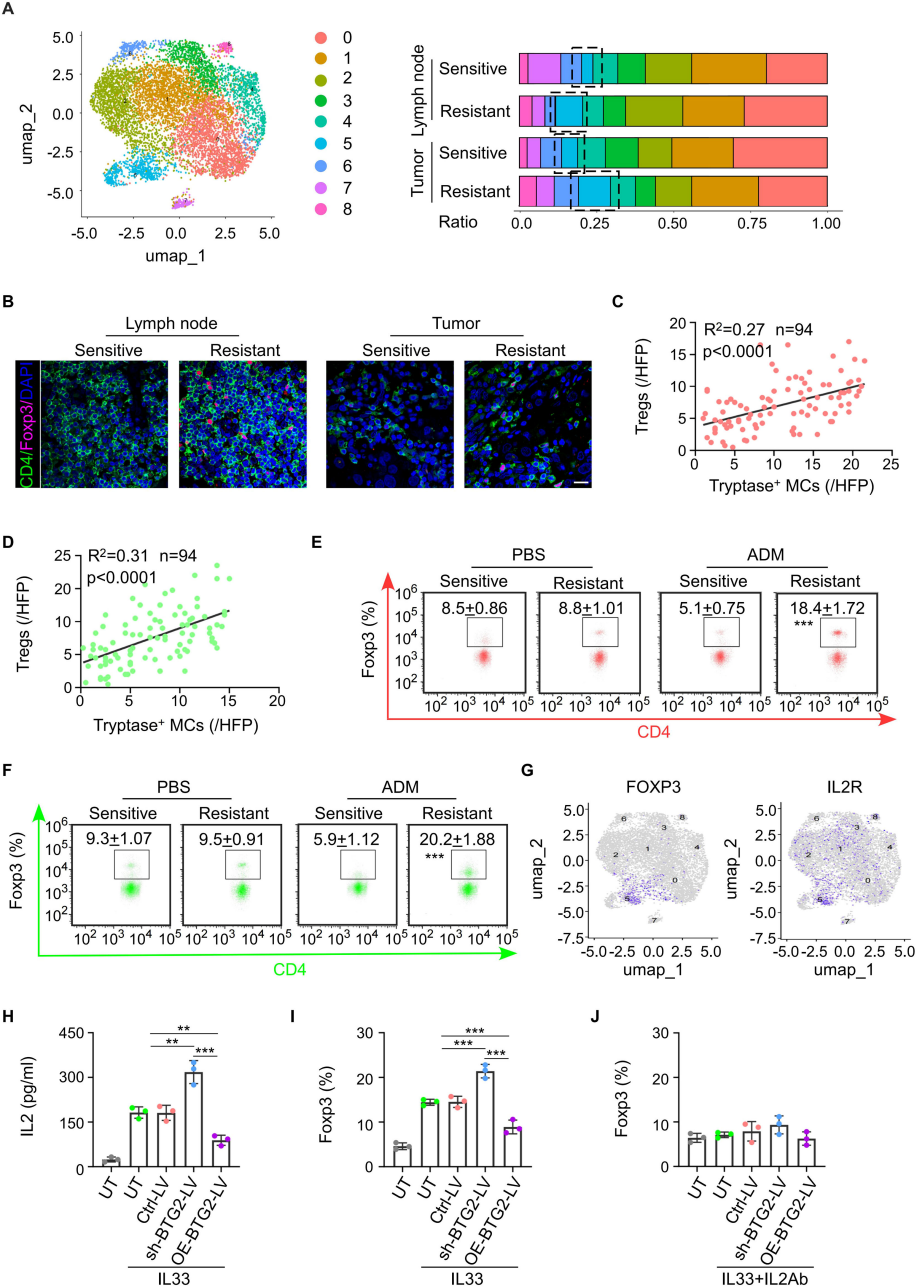
Mast cells with low BTG2 expression induce the differentiation of naïve CD4 T cells into Treg cells through IL-2, suppressing tumor immunity. **(A)** Based on single-cell sequencing results, the proportions of major immune cell types within the T cells subpopulation are displayed across different sample groups. **(B)** Representative images of Foxp3 and CD4 immunofluorescent co-staining in BC tissues (left) or TLNs tissues (right) post-neoadjuvant chemotherapy between sensitive and resistant. Scale bar, 50 μm. **(C)** The correlation between the abundance of Tryptase^+^ mast cells in tumor and the infiltration of Tregs cells in BC tissues post-neoadjuvant chemotherapy (n = 94). Pearson’s correlation coefficient (R^2^) and p values were determined by two-tailed Pearson correlation test. **(D)** The correlation between the abundance of Tryptase^+^ mast cells and Tregs in TLNs tissues of post-neoadjuvant chemotherapy (n = 94). Pearson’s correlation coefficient (R^2^) and p values were determined by two-tailed Pearson correlation test. **(E)** Flow cytometric analysis showed the population of Tregs in tumor of PDX patients with breast cancer sensitive or resistant to NAC. **(F)** Flow cytometric analysis showed the population of Tregs in TDLNs of PDX patients with breast cancer sensitive or resistant to NAC. **(G)** Gene overlays of FOXP3 and IL2R markers on t-SNE of single cells from tumor tissue and paired TDLNs tissue. **(H)** ELISA for the production of IL2 in mast cell transduced with untreat (UT) ctrl-vector (Ctrl-LV), BTG2 shRNA (shBTG2-LV) or BTG2-expressing lentivirus (OE-BTG2-LV). were treated with or without 10 ng ml^-1^ IL33. **(I)** The proportion of Foxp3^+^ cells in naive CD4^+^ T cells were co-cultured with mast cells treated with or without 10 ng ml^-1^ IL33 determined by flow cytometry. **(J)** The proportion of Foxp3^+^ cells in naive CD4^+^ T cells were co-cultured with mast cells treated with or without 10 ng ml^-1^ IL33 and 5μg ml^-1^ anti-IL2 determined by flow cytometry.

From our single-cell sequencing data, we observed a higher number of Tregs in the drug-resistant group compared to the chemotherapy-sensitive group, both within tumor tissues and lymph nodes (**Figure 4A**). This finding was confirmed through clinical pathological staining, which showed increased Tregs and decreased activated CTLs in the tumors and draining lymph nodes of the drug-resistant group (**Figure 4B**; **Supplementary Figure S4A**). Correlation analysis revealed that the number of tryptase-expressing mast cells in the tumor positively correlated with the number of mast cells in both the tumor and draining lymph nodes, and negatively correlated with the number of activated CTLs in the tumor (**Figures 4C, D**; **Supplementary Figures S4B, C**). Similarly, increased Tregs infiltration was observed in the tumor tissues and lymph nodes of chemotherapy-resistant PDX mice (**Figures 4E, F**). CTLs isolated from chemotherapy-resistant PDX tumors exhibited a weaker ability to secrete cytotoxic factors such as IFN-γ and perforin (**Supplementary Figure S4D**). Previous studies have shown that mast cells and Tregs have a certain correlation: IL-33 and activated MCs can play an anti-inflammatory or immunosuppressive negative-feedback role based on a pathway comprising IL-33-stimulated induction of activated MC-derived IL-2 leading to expansion of IL-10-producing CD4^+^ CD25^+^ Foxp3^+^ regulatory T cells (Tregs). Single-cell sequencing data also revealed significant co-localization of Foxp3 and IL2R (**Figure 4G**; **Supplementary Figure S4E**). *In vitro* experiments demonstrated that mast cells with BTG2 knockout secreted higher levels of IL-2 when stimulated by IL-33, inducing more naive CD4^+^ T cells to differentiate into Tregs. This effect could be reversed by adding IL-2 to the co-culture system (**Figures 4H–J**). Similarly, in the E0771 tumor-bearing mouse model, the application of IL-2 neutralizing antibodies reduced the increase in Tregs induced by BTG2 knockout and mitigated the CTL functional inhibition caused by BTG2 deficiency (**Supplementary Figures S4F-H**). In summary, the conclusion is that in the NAC-resistant group, the numerous mast cells, due to the lack of BTG2, are induced by IL-33 secreted by endothelial cells to produce more IL-2, thereby promoting the differentiation of Tregs, ultimately leading to suppression of anti-tumor immunity in the tumor microenvironment.

### 3.5 Tryptase produced by BTG2-insufficient mast cells induce the generation of αSAM fibroblasts promoting tumor development

**Figure 5:**
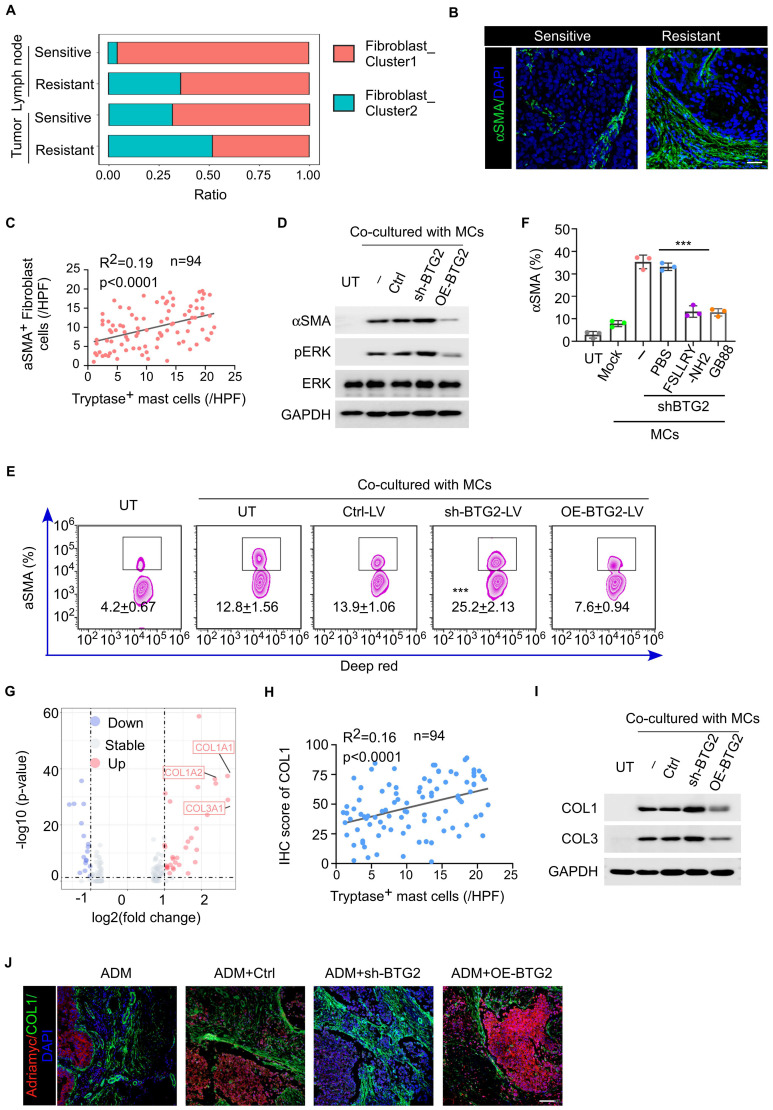
Mast cells with low BTG2 expression induces the differentiation of αSMA-positive fibroblasts and enhances their collagen secretion through secreting tryptase to promote chemoresistance. **(A)** The percentage of different cancer-associated fibroblast subsets after chemotherapy between tumor and lymph node samples. **(B)** Representative images of αSMA immunofluorescent staining in BC tissues post-neoadjuvant chemotherapy between sensitive and resistant. Scale bar, 50 μm. **(C)** The correlation between the abundance of Tryptase^+^ mast cells and αSMA^+^ cells in tumor tissues of post-neoadjuvant chemotherapy (n = 94). Pearson’s correlation coefficient (R^2^) and *p* values were determined by two-tailed Pearson correlation test. **(D)** Representative western blotting for phosphorylated/total ERK and aSMA in HFL-1 cells co-cultured with mast cells transfected with ctrl, shBTG2 or OE-BTG2. **(E)** Flow cytometry assay for the positive percentage of αSMA in HFL-1 cells co-cultured with mast cells transfected with ctrl, shBTG2 or OE-BTG2. **(F)** The positive percentage of aSMA in HFL-1 cells co-cultured with mast cells transfected with shBTG2 in culture medium with PBS, FSLLRY or GB88. **(G)** Differential gene expression analysis of fibroblast between sensitive or resistant to NAC group. The volcano plot displays log2 fold change (x-axis) versus -log10 *p*-value (y-axis) for all analyzed genes. Genes with a |log2 fold change| > 1 and a *p*-value < 0.05 are highlighted in red (upregulated) and blue (downregulated). The horizontal dashed line represents the significance threshold (*p* < 0.05), while the vertical dashed lines indicate the fold change thresholds. **(H)** The correlation between the IHC score of COL1 and the abundance of Tryptase^+^ mast cells in tumor tissues of post-neoadjuvant chemotherapy (n = 94). Pearson’s correlation coefficient (R^2^) and *p* values were determined by two-tailed Pearson correlation test. **(I)** Representative western blotting for COL1 and COL3 in HFL-1 cells co-cultured with mast cells transfected with ctrl, shBTG2 or OE-BTG2. **(J)** Representative images of COL1 and Adriamycin immunofluorescent staining in BC tissues post-neoadjuvant chemotherapy between sensitive and resistant. Scale bar, 100 μm. ***p < 0.001 by Student's t-test.

Simultaneously, we observed that compared to the NAC-sensitive sample, the NAC-resistant sample exhibited a higher percentage of αSMA^+^ fibroblast subtype (myofibroblasts) in both tumor tissue and the tumor-draining lymph nodes (**Figure 5A**). Previous research indicates that tryptase secreted by mast cells acts on PAR2 receptors on pro-fibroblasts, activating the MRK-ERK pathway to increase the expression of α-SMA, and myofibroblasts have been shown to be associated with the proliferation and migration of tumor cells. This suggests that myofibroblasts may also be involved in mast cell-induced breast cancer chemotherapy resistance. We verified that the number of myofibroblasts increased in NAC-resistant tumor tissue, and the number of activated mast cells was positively correlated with the number of myofibroblasts in tumor tissue (**Figures 5B, C**; **Supplementary Figures S5A, B**). Additionally, the activation of PAR2 receptors on fibroblasts leads to increased collagen secretion through the MRK-ERK pathway (**Figure 5D**). Mast cells with low BTG2 expression were more capable of inducing the production of αSMA^+^ fibroblasts *in vitro* or *in vivo* that could be inhibited by the addition of PAR-2 antagonists like FSLLRY-NH2 or GB83 (**Figures 5E, F**). In terms of differential gene expression, we noticed significant upregulation of genes such as COL1A1, COL1A2, and COL3A1 in fibroblasts from the resistant group compared to the sensitive group (**Figure 5G**). By co-culturing isolated primary fibroblasts with mast cells expressing low levels of BTG2 under IL-33-induced degranulation for 24 hours, we measured the concentration of collagen in the culture medium. Mast cells with low BTG2 expression were more capable of inducing fibroblasts to produce more collagen, a process that could be inhibited by the addition of PAR-2 antagonists like FSLLRY-NH2 or GB83, consequently halting collagen production (**Figures 5H, I**; **Supplementary Figures S5C-E**). Additionally, in a mouse PDX model, staining of tumor slices from the resistant group revealed higher secretion of type I collagen by fibroblasts, accompanied by fewer infiltrating ADMs into the tumor core. Exogenous injection of shBTG2 mast cells led to increased collagen in mouse tumor tissues (**Figure 5J**). These pieces of evidence indicate that the lack of BTG2 in mast cells leads to extensive expression of tryptase, influencing the differentiation of fibroblasts into αSMA-positive myofibroblasts. The activation of PAR2 receptors on fibroblasts results in collagen secretion, increasing tumor stiffness, forming a robust niche that hinders the entry of chemotherapy drugs, consequently causing chemoresistance.

### 3.6 Radiotherapy can rescue the expression of BTG2 in mast cells and thereby enhance the efficacy of chemotherapy

**Figure 6:**
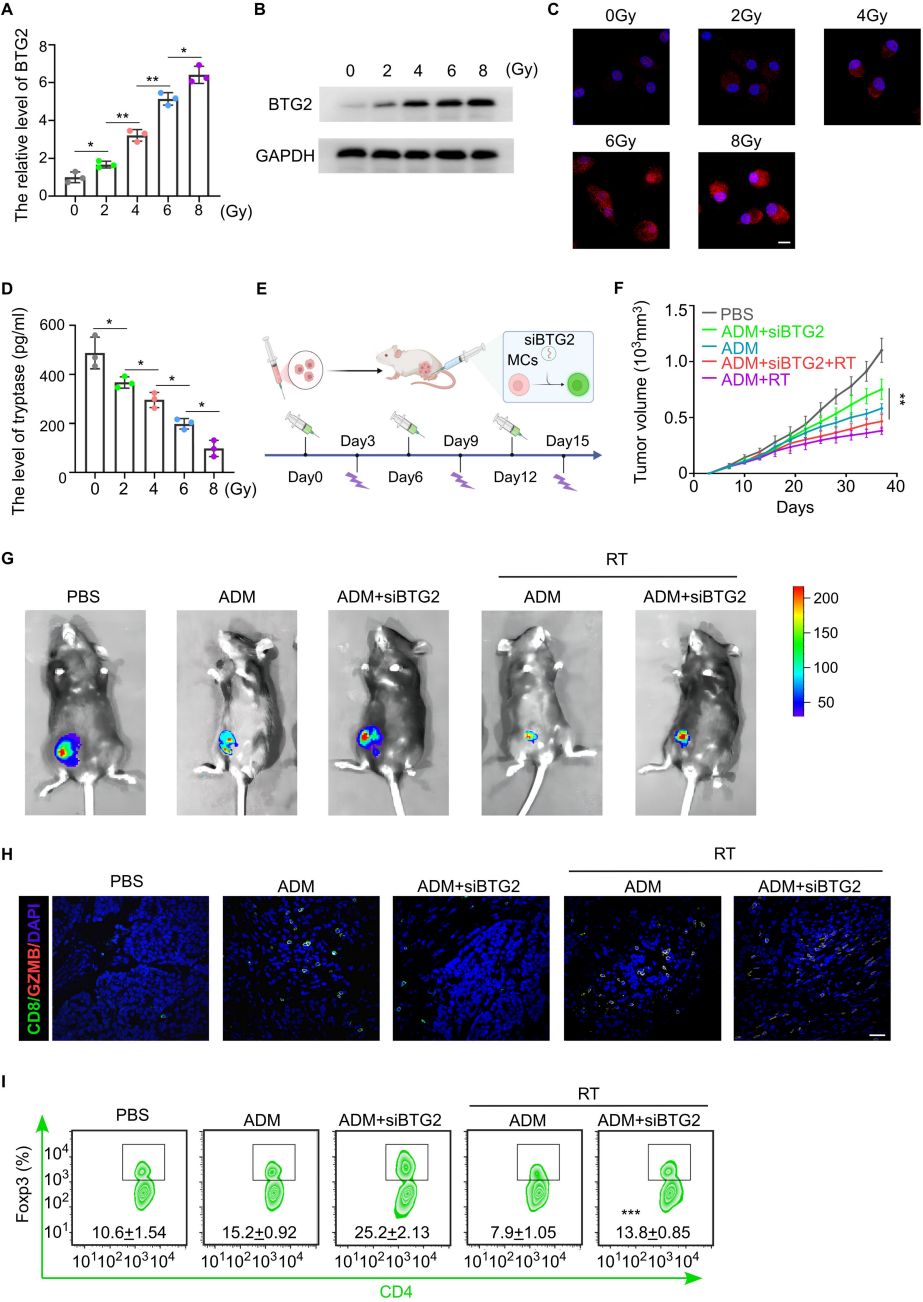
Radiation therapy can improve chemotherapy resistance in breast cancer by upregulating the expression of BTG2 in mast cells. **(A)** The fold change of BTG2 mRNA expression in mast cells irradiated with 0, 2, 4, 6,8 Gy. **(B)** Western blot assay the expression level of BTG2 in mast cells irradiated with 0, 2, 4, 6,8 Gy. **(C)** Representative images of BTG2 immunofluorescent staining in mast cells irradiated with 0, 2, 4, 6,8 Gy. Scale bar, 10 μm. **(D)** The ELISA assay for the expression level of Tryptase in mast cells irradiated with 0, 2, 4, 6,8 Gy. **(E-I)** Luciferase-E0771 tumor-bearing mice were externally infused with shBTG2 mast cells. Once the tumors grew to 150mm³, the mice were randomly assigned to different experimental groups and received either chemotherapy and radiotherapy or no treatment, with a 6-day cycle. **(E)** Schematic of tumor-bearing mouse model experiment. **(F)** The tumor growth curve. **(G)** The Representative bioluminescent images showing the tumor growth. **(H)** Representative images of GZMB and CD8 immunofluorescent co-staining in tumor tissue. Scale bar, 50 μm. **(I)** Flow cytometry assay for the positive percentage of Foxp3 in CD4 cells isolated from TLNs. *p < 0.05, **p < 0.01, ***p < 0.001 by Student's t-test.

Previous experimental studies have demonstrated that a deficiency of BTG2 in mast cells promotes an immunosuppressive state within the tumor microenvironment (TME) and contributes to chemotherapy resistance. Given these findings, we aimed to identify a therapeutic strategy to enhance the efficacy of breast cancer chemotherapy by upregulating BTG2 expression in mast cells. Recent research suggests that low-dose radiotherapy (LDRT) not only serves as a direct anti-tumor treatment but also exerts potent immunomodulatory effects (30). Notably, BTG2, a radiation-responsive gene, has been reported to undergo dose-dependent upregulation in tumor cells following radiotherapy. To explore whether radiation could similarly regulate BTG2 expression in mast cells, we exposed mast cells to graded doses of ionizing radiation (IR) *in vitro*. The results demonstrated a corresponding increase in BTG2 mRNA and protein expression levels in a dose-dependent manner (**Figures 6A–C**). Furthermore, when stimulated with IL-33, irradiated mast cells exhibited a dose-dependent reduction in the secretion of tryptase and IL-2 (**Figure 6D**; **Supplementary Figure S6A**), suggesting an attenuation of their pro-inflammatory and immunosuppressive functions. To further investigate the functional consequences of this radiation-induced BTG2 upregulation, we conducted co-culture experiments. Naïve CD4+ T cells co-cultured with irradiated mast cells exhibited a dose-dependent reduction in the proportion of Tregs, highlighting a potential shift toward an anti-tumor immune response. A similar trend was observed when fibroblast precursor cells were co-cultured with irradiated mast cells, where the proportion of Tregs and the secretion of collagen fibers by fibroblasts inversely correlated with the IR dose gradient (**Supplementary Figures S6B–D**).

To validate these findings *in vivo*, we infused tumors with exogenous BTG2-knockdown mast cells and assessed chemotherapy sensitivity following IR treatment. Notably, tumors in mice receiving IR treatment displayed significantly smaller sizes and reduced lymph node metastases, suggesting enhanced chemotherapy efficacy in the presence of BTG2-knockdown mast cells (**Figures 6E–G**; **Supplementary Figure S6E**). Flow cytometric analysis of tumor tissues further revealed increased infiltration of activated cytotoxic T lymphocytes (CTLs) and a reduction in Tregs in the IR-treated group (**Figures 6H–J**), supporting the notion that LDRT promotes an anti-tumor immune response through mast cell modulation. Collectively, these findings indicate that LDRT enhances chemotherapy sensitivity in breast cancer by upregulating BTG2 expression in mast cells, thereby reprogramming the TME toward a more immunostimulatory state. This highlights the potential of LDRT as a novel immunomodulator and chemosensitizer, offering a promising therapeutic strategy to improve clinical outcomes in breast cancer treatment.

## 4 Discussion

In this study, we identified a mast cell subpopulation associated with resistance to neoadjuvant chemotherapy, characterized by low BTG2 expression, through scRNA sequencing of primary breast cancer samples and paired metastatic lymph nodes. Mast cells with low BTG2 expression exhibit several key features that collectively contribute to chemotherapy resistance. First, we observed that these mast cells possess enhanced migratory ability, enabling them to be more readily attracted from the periphery to tumors and their draining lymph nodes by chemokines such as CCL5. Notably, low-BTG2-expressing mast cells produce higher levels of IL-2, which, under IL-33 stimulation, promotes the differentiation of naive CD4^+^ T cells into Tregs especially in lymph nodes. Additionally, mast cells with low BTG2 expression could induce an increased proportion of αSMA-positive myofibroblasts, which enhance tumor invasiveness and metastatic potential by altering the glycosylation patterns of tumor cells. On the other hand, low-BTG2-expressing mast cells stimulate fibroblasts to secrete collagen, forming a rigid tumor niche that impedes the penetration of chemotherapeutic drugs, further exacerbating resistance to treatment.

Mast cells play a dual role in cancer, acting as both promoters and inhibitors of tumor progression (31). They promote growth and metastasis by enhancing angiogenesis through VEGF release, remodeling the extracellular matrix with proteases, and creating an immunosuppressive environment through recruitment of Tregs and secretion of cytokines such as IL-10 and TGF-β (32). Conversely, mast cells also play antitumor roles by stimulating immune responses, recruiting cytotoxic T cells and NK cells, secreting cytotoxic mediators such as TNF-α and ROS, and enhancing immune surveillance through dendritic cell maturation and tumor antigen presentation (33, 34). Here, we found that mast cells, characterized by low expression of BTG2, were enriched in the *in situ* tumor tissue and draining lymph nodes of patients with neoadjuvant chemotherapy-resistant breast cancer. Clinical pathological section staining analysis confirmed that mast cells with low BTG2 expression were negatively correlated with chemotherapy efficacy. This clinical phenomenon was then reproduced in animal experiments by establishing a chemotherapy-resistant PDX mouse model. In addition, exogenous infusion of mast cells expressing BTG2 was used to intervene, and changes in chemotherapy efficacy were observed, further confirming that this group of BTG2 mast cells is one of the culprits causing chemotherapy resistance.

The BTG2 gene (B-cell translocation gene 2), as a tumor suppressor gene, plays an important role in the occurrence and development of various tumors (35). It mainly inhibits tumor progression by regulating the cell cycle, promoting cell apoptosis, and affecting DNA damage repair (36), and could be a good target for drug design for cancer such as ERH (Enhancer of Rudimentary Homolog) (37). BTG2 has been proven to be downregulated in breast cancer, liver cancer, lung cancer, colorectal cancer and other tumors, and is closely related to the malignancy, recurrence and metastasis of the tumor (38–41). Recent studies have focused on BTG2 is positively correlated with the stable state of various immune cells, such as T cells and B cells. In addition, the expression level of BTG2 is negatively correlated with activated mast cells in lung cancer had been identified (42). We verified the negative relationship between BTG2 and mast cell activation in breast cancer through our sc-RNA sequencing data and TCGA database, and confirmed that mast cells lacking BTG2 were more prone to produce the activation marker trypsin, which activated the PAR2 receptor-MEK pathway on precursor fibroblasts to induce myofibroblast subpopulations and the secretion of collagen. Serum tryptase showed potential as a tumor biomarker, particularly in mast cell-related neoplasms like systemic mastocytosis, where it correlates with tumor burden (43). Elevated levels are also observed in solid tumors (e.g., breast cancer and colo-rectal cancer) and associated with poor prognosis (44, 45). Our preliminary data suggest a correlation between tryptase levels and BTG2 expression in tumor tissues and in mast cells, but the serum tryptase specificity is limited by non-neoplastic conditions such as allergies. There may be potential utility of serum tryptase levels as a non-invasive biomarker for BTG2-low mast cell and tumor prognosis, further validation in a larger cohort is needed. Previous studies have shown that IL-33 secreted by endothelial cells induce the generation of Tregs by activating mast cells via producing IL-2 (46). Consistent with these findings, our study identified this mechanism as a key pathway through which mast cells with low BTG2 expression contribute to immunosuppression. In addition, as previously found in tumor cells, BTG2 can maintain more Src in a reduced state by reducing the level of ROS in mitochondria, thereby reducing the phosphorylation of FAK molecules. We verified that the migration ability of mast cells with knocking down the expression of BTG2 was enhanced *in vitro* and *in vivo* experiments. Our study fills the gap in the research field of the BTG2 gene in mast cells and provides a new specific target molecule for the development of chemotherapy resistance strategies for breast cancer.

The latest research progress in breast surgery primarily focuses on “de-escalation” (47). There remains controversy over whether axillary lymph node dissection (ALND) can be waived for patients with isolated tumor cells (ITCs) in sentinel lymph nodes (SLNs) post-neoadjuvant therapy (NAT) or for those achieving clinical complete response (CR) after NAT (48). Four key studies have explored axillary lymph node de-escalation strategies. Data from the SOUND study and results presented at the 2024 San Antonio Breast Cancer Symposium (SABCS) from the INSEMA trial suggest that, for certain breast cancer patients meeting specific criteria-including older age, smaller tumor size, preoperative tumor size <2 cm, and histological grade 1-2 HR^+^/HER2^-^ tumors-waiving ALND does not significantly affect survival, prognosis, or the risk of local recurrence (49, 50). In our research, we demonstrated that low BTG2 expression in mast cells within lymph nodes indicates an immunosuppressive microenvironment, which may correlate with an increased likelihood of distant tumor recurrence. Radiotherapy has been shown to reverse the immunosuppressive state of these “bad lymph nodes,” potentially enhancing the safety of omitting ALND in eligible patients (51). For patients with negative axillary lymph node ultrasound findings, the number of mast cells and BTG2 expression levels in tumor tissue can be assessed to determine whether additional radiotherapy is needed to improve the immune suppression in micrometastatic lymph nodes. By assessing the number of mast cells and BTG2 expression levels in SLNs, it may be possible to determine whether the remaining lymph nodes are sufficiently safe. Furthermore, intensified radiotherapy could serve as a compensatory treatment in cases of borderline eligibility for ALND omission. In conclusion, our findings suggest that low-dose radiotherapy into treatment regimens may further enhance chemotherapy efficacy while supporting lymph node preservation strategies, paving the way for innovative and precise treatment approaches to improve patient outcomes.

This study has several limitations. Firstly, the sample size is relatively small, as we conducted scRNA-seq on only eight patients, which restricts the generalizability of our conclusions. Secondly, we did not utilize gene-edited mice with specific BTG2 knockout in mast cells to perform targeted gene knockout experiments. Instead, we relied on exogenous infusion of BTG2-knockdown mast cells, which may not fully capture the natural differentiation, transformation, and activation of mast cells *in vivo*. Thirdly, there is a lack of long-term survival and prognosis analysis for breast cancer patients with low BTG2 expression in mast cells, limiting the broader applicability of our findings.

In summary, we identified a specific subpopulation of mast cells associated with neoadjuvant resistance, characterized by low expression of BTG2, elucidated how mast cells with low BTG2 expression were significantly recruited to tumors and lymph nodes, and detailed the specific mechanism by which they jointly lead to chemotherapy resistance by affecting the subtype differentiation of T cells and fibroblasts. Our study provides profound insights into how to improve breast cancer chemotherapy resistance in the future and paves the way for personalized treatment of breast cancer patients.

## Data Availability

The original contributions presented in the study are included in the article/**Supplementary Materials**. Further inquiries can be directed to the corresponding authors.
