# Supplementary material for: BTG2-deficient mast cells remodel the tumor and tumor-draining lymph node microenvironment leading to chemotherapy resistance in breast cancer
Source: Front Immunol. 2025 Apr 17;16:1562700. doi: 10.3389/fimmu.2025.1562700 (PMC12043456; doi:10.3389/fimmu.2025.1562700)
Supplement: Supplementary file 1 [file DataSheet1.docx]

Supplementary Material

# Supplementary Figures and Tables

## Supplementary Figures

## Supplementary Table

## Supplementary Graph


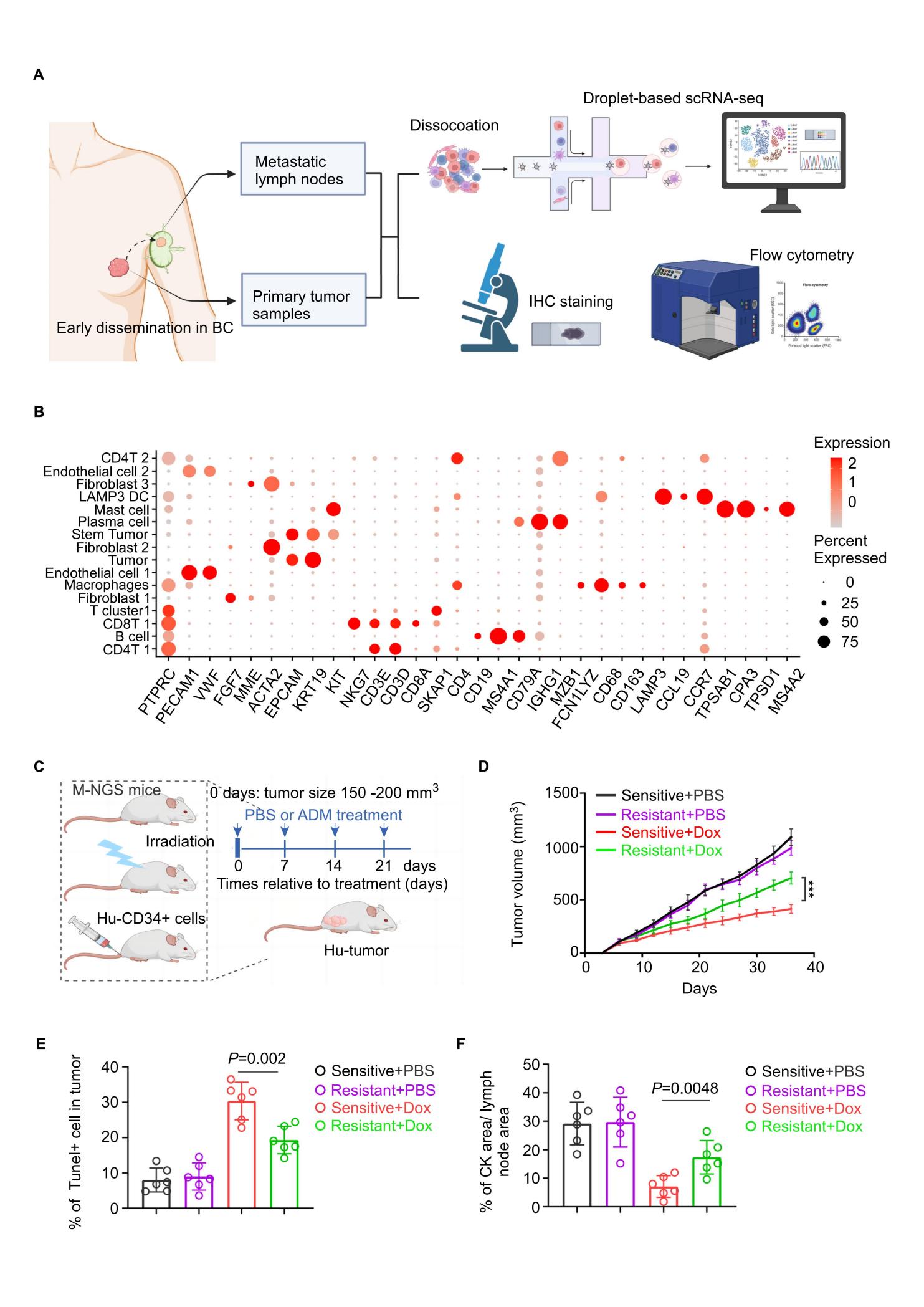


**Figure S1. Breast cancer patients with chemoresistance exhibit a significantly higher density of mast cells in both primary tumor sites and draining lymph nodes.**

**(A)** Graphic overview of this study design. Tumor and paired lymph node tissue from four breast cancer patients were processed into single-cell suspension and unsorted cells were used for scRNA-seq with 10x Genomics. Tumor slides were processed to obtain by 10x Genomics Visium. The following integrated analysis of cytological experiments and IHC staining is described in squares.

**(B)** Bubble plots of the marker genes expressed in the major cell types. Dot color reflects expression level and dot size represents thepercent of cells expressing marker genes in different cell types.

**(C-D)** Fresh tumor tissues obtained from BC tissues after NAC with chemotherapy-sensitive or -resistant response were cut into 2-3 mm³slices for implantation into the mammary fat pad of Irradiate NSG mice injected human peripheral blood-derived CD34^+^ cells. When tumor volumes reached approximately 150-200mm^3^, mice received intraperitoneal administration of ADM at 5 mg/kg once a week. PBS served as treatment control.

**(C)** Schematic illustration of the treatment process of breast cancer PDX model mice:The PDX models are established by transplanting fresh tumor tissue resected from human breast cancer sensitive or resistant to NAC into mice.

**(D)** Tumor growth curves of PBS- or ADM-treated sensitive or resistant PDX syngrafts.

**(E)** Immunofluorescent quantitation of Tunel expressing tumor cells in tumor from PBS- or ADM-treated sensitive or resistant PDX syngrafts.

**(F)** Immunofluorescent quantitation of tumor cells in breast cancer TLN from PBS- or ADM-treated sensitive or resistant PDX syngrafts.

**
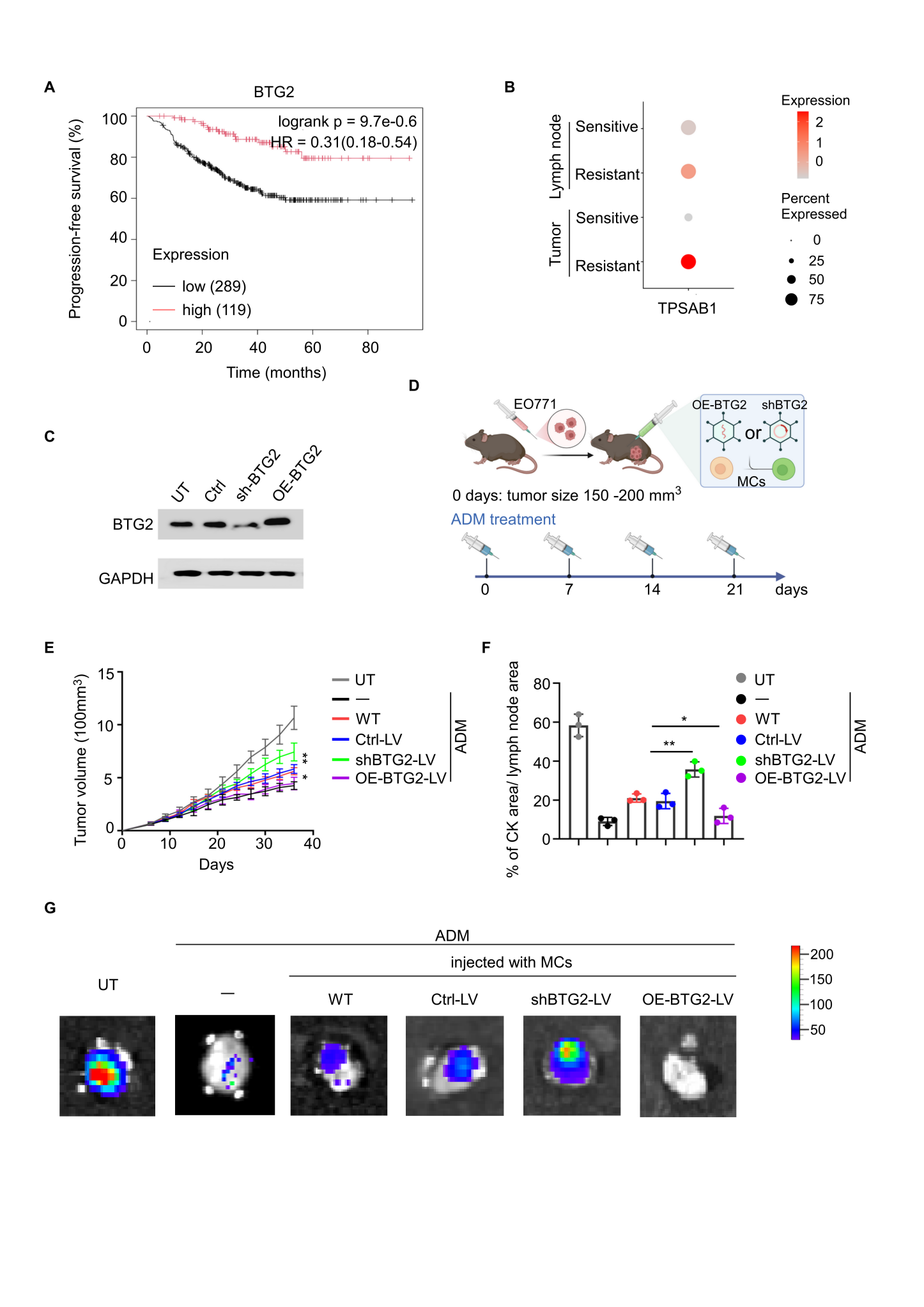
**

**Figure S2. The mast cells are characterized by low expression of BTG2, and exogenous infusion of BTG2sh mast cells reduce the efficacy of chemotherapy.**

**(A)** The progression-free survival analysis of lymph node-positive patients undergoing neoadjuvant chemotherapy with high (n=119) and low (n=289) expression of BTG2 from TCGA database.

**(B)** The expression level of the tryptase gene (TPSAB1) in mast cells across different samples.

**(C)** Western blot of BTG2 expression in mast cells were transfected without (UT) or with ctrl, sh-BTG2 or OE-BTG2 plasmid.

**(D-G)** Mice received inoculation of EO771 cells were randomly divided into six groups for different treatments. When tumor volume reached 150-200mm^3^, mice were administrated with PBS or 5 mg/kg ADM once a week, in the presence or absence of tail vein injection of 1×10^4^ mast cells transfected without (UT) or with ctrl, sh-BTG2 or OE-BTG2 plasmid.

**(D)** Schematic illustration of the treatment process.

**(E)** Tumor growth curves.

**(F)** The quantification of CK+ cell in TLN tissue.

**(G)** Immunofluorescent quantitation of tumor cells in BC draining-lymph node.


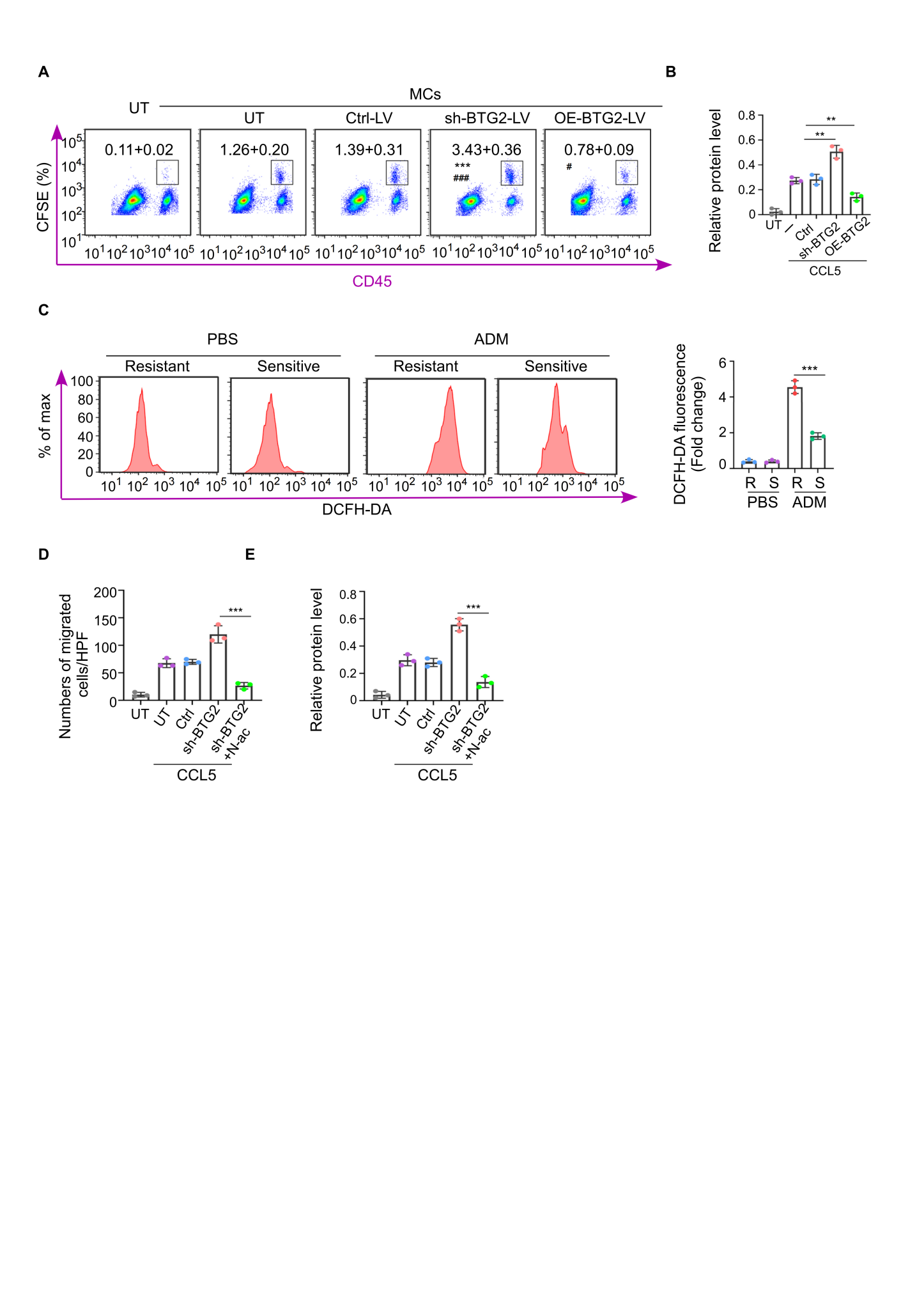


**Figure S3.** **Mast cells with low BTG2 expression have stronger migration ability.**

**(A)** Representative flow cytometry images for CFSE-label cells in tumor tissues of EO771 tumor-bearing mice in the presence or absence of tail vein injection of 1×10^4^ CESE-label mast cells transfected without (UT) or with ctrl, sh-BTG2 or OE-BTG2 plasmid.

(B) Relative FAK phosphorylation protein levels quantified using ImageJ (mean ± s.e.m., n= 3 independent experiments, protein levels were normalized using GAPDH as the loading control) correspond to Figure 3E.

**(C)** Representative flow cytometry images for ROS in tumor tissues of E0771 tumor-bearing mice in the presence or absence of tail vein injection of 1×10^4^ CESE-label mast cells transfected without (UT) or with ctrl, sh-BTG2 or OE-BTG2 plasmid.

**(D-E)** Mast cells transduced with untreat (UT) ctrl-vector (Ctrl-LV), BTG2 shRNA (shBTG2-LV) or BTG2-expressing lentivirus (OE-BTG2-LV). were treated with or without 50 ng ml^-1^ CCL5 or with 5mM N-ac.

**(D)** The numbers of migration cells.

**(E)** Relative FAK phosphorylation protein levels quantified using ImageJ (right) (mean ± s.e.m., n= 3 independent experiments, protein levels were normalized using GAPDH as the loading control).


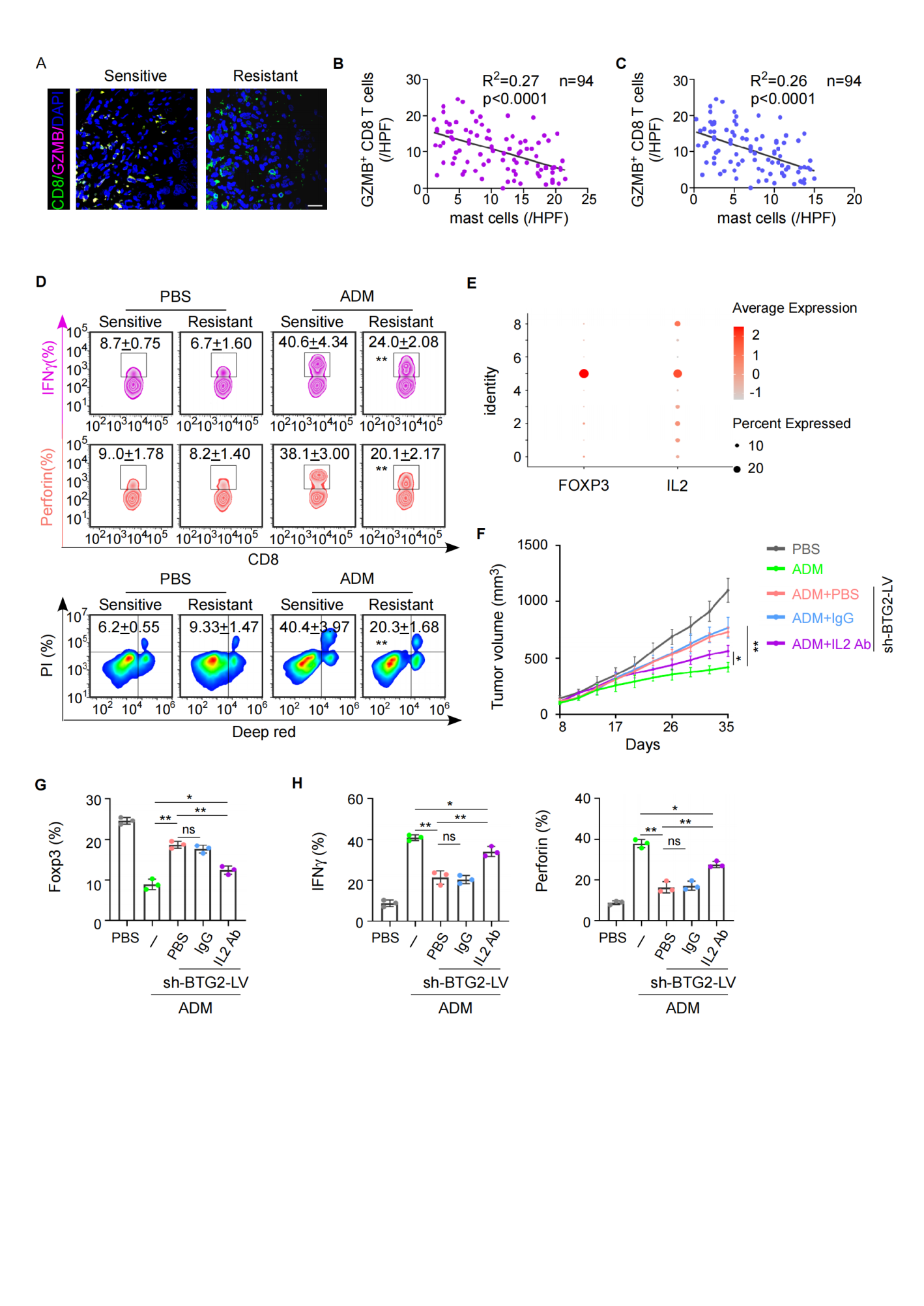


**Figure S4. Mast cells with low BTG2 expression induce the differentiation of naive CD4 T cells into Treg cells through IL-2, suppressing tumor immunity.**

**(A)** Representative images of GZMB and CD8 immunofluorescent co-staining in BC tissues post-neoadjuvant chemotherapy between sensitive and resistant. Scale bar, 50 μm.

**(B)** The correlation between the the abundance of GZMB^+^ CD8^+^ T cells in tumor and the infiltration of Tryptase^+^ mast cells in tumor post-neoadjuvant chemotherapy (n = 94). Pearson’s correlation coefficient (R^2^) and p values were determined by two-tailed Pearson correlation test.

**(C)** The correlation between the the abundance of GZMB^+^ CD8^+^ T cells in tumor and the infiltration of Tryptase^+^ mast cells in TLNs tissues post-neoadjuvant chemotherapy (n = 94). Pearson’s correlation coefficient (R^2^) and p values were determined by two-tailed Pearson correlation test.

**(D)** The CD8^+^ T cells isolated from TLN tissues of PBS- or ADM-treated sensitive or resistant PDX syngrafts were treated co-cultured with EO771 cell lysates for 12h. The percentage of IFN^-^γ^+^ (up) and perforin^+^ (median) of treated CD8^+^ T cells as shown. Then treated CD8^+^ T cells were co-cultured with EO771 cell, and tumor specific killing of CD8^+^ T cells (low) were shown.

**(E)** Dot plot showing the co-expression of two genes based on single-cell sequencing analysis.

**(F-H)** Mice received inoculation of EO771 cells when tumor volume reached 100-150mm^3^, mice were administrated with PBS or 5 mg/kg ADM once a week, in the presence of tail vein injection of 1×10^4^ mast cells transfected with sh-BTG2 plasmid. The mice were then randomly divided into groups and subjected to intraperitoneal injection of PBS, IgG, or IL-2 antibody.

**(F)** Tumor growth curves.

**(G)** The percentage of Foxp3^+^ cells in tumor infiltrating CD4^+^ T cells were evaluated by flow cytometry.

**(H)** The IFN-γ (left) and perforin (right) and expression of tumor-infiltrating CD8^+^ T cell were evaluated by flow cytometry.

**
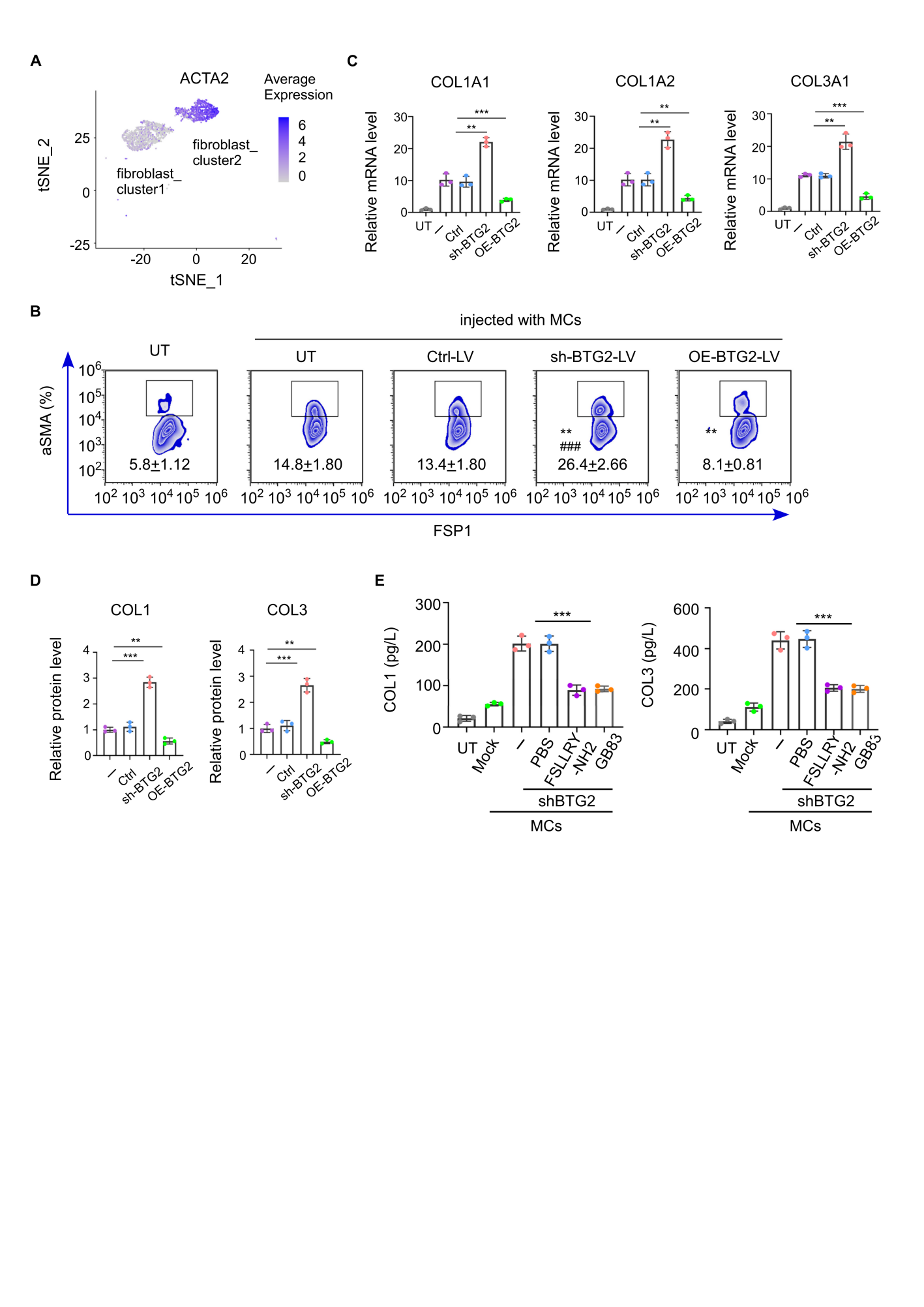
**

**Figure S5. Mast cells with low BTG2 expression induces the differentiation of αSMA-positive fibroblasts and enhances their collagen secretion through secreting tryptase to promote chemoresistance**

**(A)** The expression level of the ACTA2 gene in between fibroblast cluster1 and fibroblast cluster2.

**(B)** Flow cytometry assay for the positive percentage of αSMA in CAFs isolated tumor tissues , mice under exogenous injection with mast cells transfected with ctrl, sh-BTG2 or OE-BTG2.

**(C)**The mRNA level of COL1A1, COL1A2 and COL3A1 in HFL-1 cells co-cultured with mast cells transfected with ctrl, sh-BTG2 or OE-BTG2.

**(D)** Relative COL1 and COL3 protein levels quantified using ImageJ (mean ± s.e.m., n= 3 independent experiments, protein levels were normalized using co-cultured with UT mast cells group as the loading control).

**(E)** The level of COL1 and COLA3 in HFL-1 cells co-cultured with mast cells transfected with shBTG2 in culture medium with PBS, FSLLRY-NH2 or GB83.


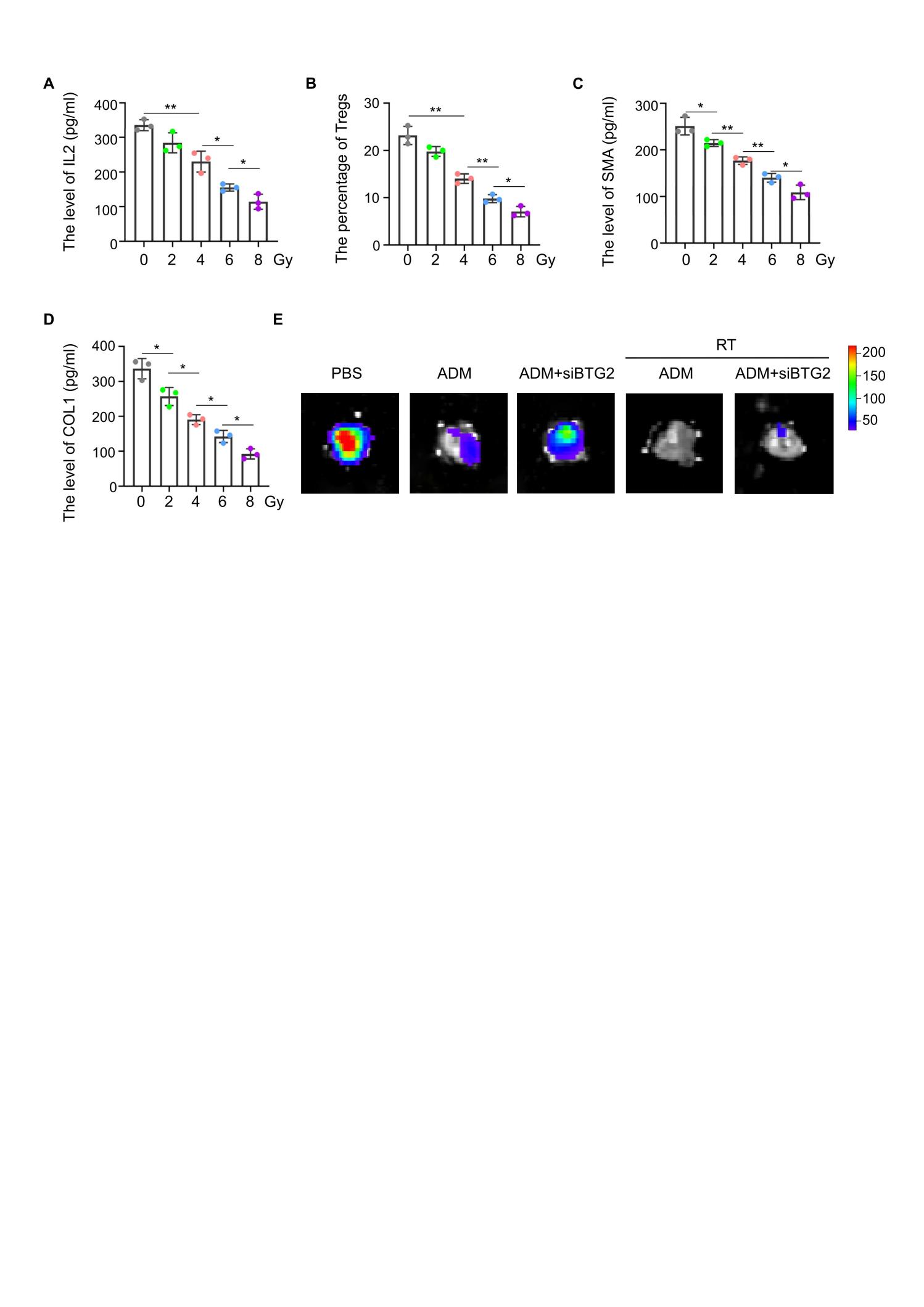


**Figure S6. Radiation therapy can improve chemotherapy resistance in breast cancer by upregulating the expression of BTG2 in mast cells.**

**(A)** The ELISA assay for the expression level of IL2 in mast cells irradiated with 0, 2, 4, 6, 8 Gy.

**(B)** Flow cytometry assay for the positive percentage of Foxp3 in CD4 cells co-cultured with mast cells irradiated with 0, 2, 4, 6, 8 Gy.

**(C)** The ELISA assay for the expression level of aSMA in the supernatant of HFL-1 cells co-cultured with mast cells irradiated with 0, 2, 4, 6, 8 Gy.

**(D)** The ELISA assay for the expression level of COL1 in the supernatant of HFL-1 cells co-cultured with mast cells irradiated with 0, 2, 4, 6, 8 Gy.

**(E)** Luciferase-E0771 tumor-bearing mice model experiment as Figure 6E descripted, and the Representative bioluminescent images showing the lymphatic metastasis.

**1.2 Supplementary Table**

Supplementary Table

|  | age(year) | tumor size | Nodal status | Grading | Hormone receptor | HER2 status | Distant metastases |
| --- | --- | --- | --- | --- | --- | --- | --- |
| patient1 | 40 | T2 | Positive | Moderately differentiated(G2) | Negative | Positive | M0 |
| patient2 | 39 | T2 | Positive | Moderately differentiated(G2) | Negative | Positive | M0 |
| patient3 | 42 | T3 | Positive | Moderately differentiated(G2) | Negative | Negative | M0 |
| patient4 | 38 | T3 | Positive | Moderately differentiated(G2) | Negative | Negative | M0 |

**1.3 Supplementary Graph**


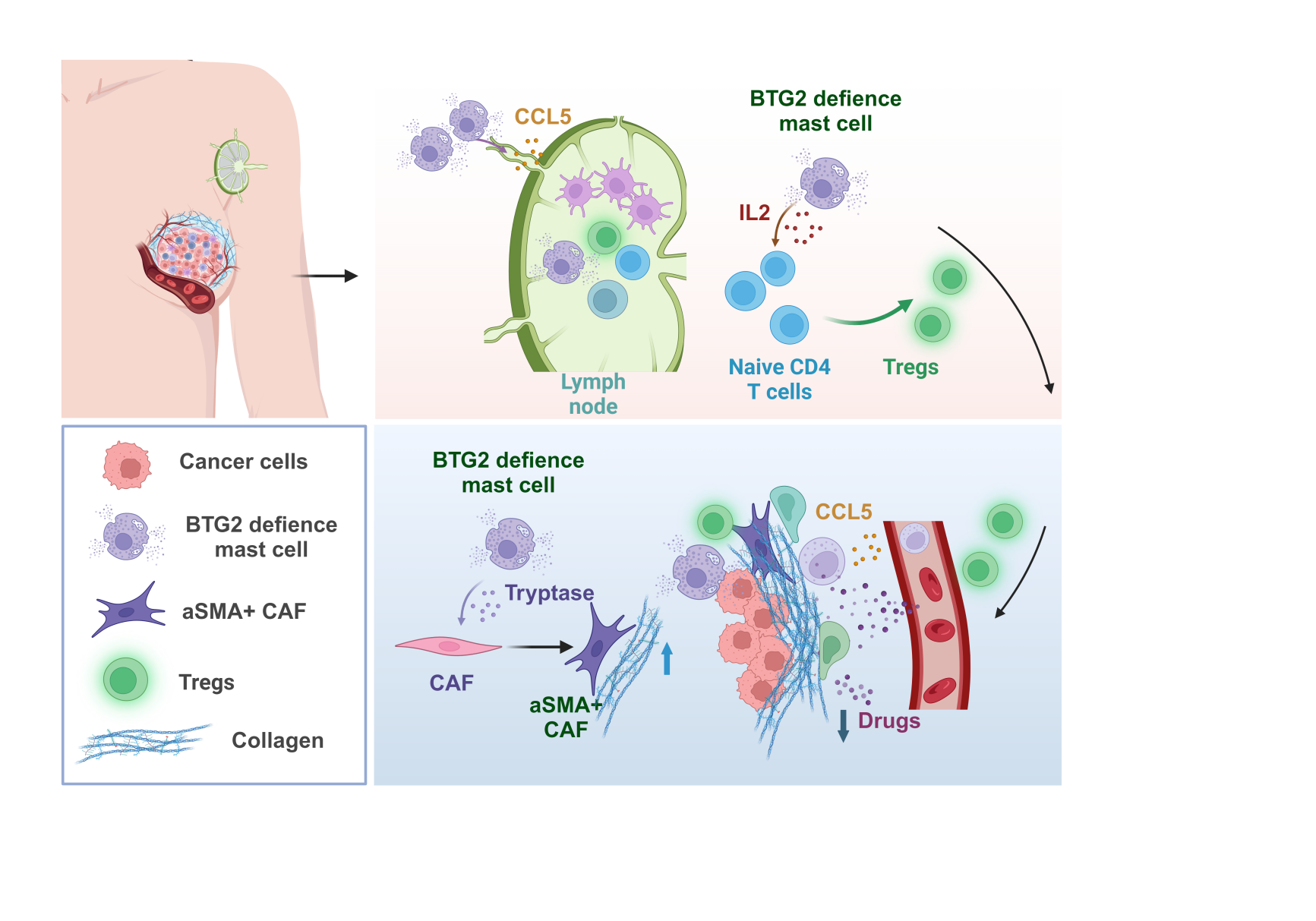
**Graphical Abstract**
